# Transcriptomic Analysis of Fish Hosts Responses to Nervous Necrosis Virus

**DOI:** 10.3390/pathogens11020201

**Published:** 2022-02-03

**Authors:** Dimitra K. Toubanaki, Antonia Efstathiou, Evdokia Karagouni

**Affiliations:** Immunology of Infection Group, Department of Microbiology, Hellenic Pasteur Institute, 11521 Athens, Greece; toniaef@pasteur.gr

**Keywords:** betanodaviruses, viral nervous necrosis, viral encephalo-retinopathy, transcriptomic, RNA-Seq, host-pathogen interaction

## Abstract

Nervous necrosis virus (NNV) has been responsible for mass mortalities in the aquaculture industry worldwide, with great economic and environmental impact. The present review aims to summarize the current knowledge of gene expression responses to nervous necrosis virus infection in different fish species based on transcriptomic analysis data. Four electronic databases, including PubMed, Web of Science, and SCOPUS were searched, and more than 500 publications on the subject were identified. Following the application of the appropriate testing, a total of 24 articles proved eligible for this review. NNV infection of different host species, in different developmental stages and tissues, presented in the eligible publications, are described in detail, revealing and highlighting genes and pathways that are most affected by the viral infection. Those transcriptome studies of NNV infected fish are oriented in elucidating the roles of genes/biomarkers for functions of special interest, depending on each study’s specific emphasis. This review presents a first attempt to provide an overview of universal host reaction mechanisms to viral infections, which will provide us with new perspectives to overcome NNV infection to build healthier and sustainable aquaculture systems.

## 1. Introduction

Aquaculture has been evolved from playing a relatively minor role to a major part of the global food system. Blue revolution is based on the aquaculture industry, highlighting its significant role as a vital and very productive agricultural activity [1]. In fact, aquaculture produced more than 82.1 million tons (Mt) of fish, including around 425 farmed species in 2018, with an increasing trend in fish farming. The Food and Agriculture Organization (FAO) predicts that global fish production will reach 204 Mt in 2030 [2]. However, pathogens, parasites, pests, pollution, harmful algal blooms, and climate change cause challenging issues for the industry with negative impact [3,4]. Especially diseases of viral etiology have been wreaking havoc in the aquaculture industry, sometimes wiping out entire stocks within days of onset of infection [5]. Host-pathogen interactions concerning viral infections of teleost fish are under intense investigation since the effectiveness of immune defense mechanisms is tightly related to the host health [6], and it is anticipated that the systematic study of -omics datasets through a systems biology approach will enable scientists to describe the complexity and characteristics of interactions in the host-pathogen network, leading to the identification of new biomarkers and drug targets for fish diseases [4]. Therefore, transcriptome analysis is an essential tool for optimum understanding of the underlying mechanisms of the host response to a pathogen.

A transcriptome is the complete set of transcripts in a cell, tissue, or organ under a specific developmental stage or physiological condition [7]. Transcriptomic analysis reveals a snapshot of the cellular processes that are active or dormant under specific conditions. Transcriptomic-related technologies have been evolved rapidly (within a decade) from hybridization-based microarrays to RNA sequencing (RNA-seq), based on next-generation sequencing (NGS) platforms. Microarray technology revolutionized the field when first introduced, but it has several limitations, including the requirement of prior knowledge of gene sequences from the studied organism, artifact occurrence caused by cross-hybridization, high background noise, signal saturation issues, laborious methodology for tag-based microarray quantification methods, the requirement of large quantities of input RNA, high cost, low accuracy on quantification of spliced isoforms, and inability to discover novel genes [8]. Nowadays, the RNA-seq technology has replaced microarrays due to advantages such as the ability to measure gene expression of low-abundance transcripts and isoforms de novo based on reading counts with great confidence, the opportunity to generate whole transcriptomes in almost any non-model organisms of interest without prior genome information, its relatively lower cost and better coverage/resolution compared to the previously used methods for transcriptome analysis. In the aquaculture research field, RNA-seq has mostly been used for fish immunology studies in order to understand the underlying mechanisms and pathways of host-pathogens interactions during the course of infection. Moreover, it has been applied in studies for embryo and larvae development, dietary and toxic and environmental stress effects, and the discovery of novel transcripts [9,10,11].

## 2. Nervous Necrosis Virus

Viral nervous necrosis (VNN) or vacuolating encephalopathy and retinopathy (VER) or encephalomyelitis is a disease of viral aetiology, causing high morbidity and mortality with rates up to 100% in some species [12]. The clinical symptoms of NNV infection depend on the fish species, biological stage, phase of the disease and temperature, and include abnormal swimming behavior (spiral swimming, whirling, horizontal looping or darting), loss of appetite, swim bladder hyperinflation, and coloration abnormalities (pale or dark), resulting eventually in the death of infected hosts [13]. Histopathological analysis revealed extensive vacuolation and neural degeneration of the brain and the retina, combined with necrosis of the central nervous system (CNS) [14]. Since NNV mostly affects the nervous system, its role in fish neuroimmune communication is expected to be crucial. Although the nervous system and the immune system were believed to work independently, there are indications that crosstalk between neurons and immune cells occurs both in the CNS and the periphery [15,16]. However, such studies in fish are still limited. Both horizontal and vertical transmission has been demonstrated in various fish species [17]. Persistent or carrier infection may develop to an acute phase with biological and environmental stress factors and facilitate the vertical or horizontal transmission of the nodavirus [18].

The causing agent of VNN is nervous necrosis virus (NNV) or fish Betanodavirus, which is a pathogen affecting more than 120 different species from marine and freshwater environments [12,19]. NNV is an icosahedral, non-enveloped virus with a diameter of ~25 nm, and its genome is formed by two single-stranded, 5′-capped but not polyadenylated, positive-sense RNA molecules [13], i.e., RNA 1 (3.1 kb), which directs the RNA-dependent RNA polymerase synthesis (1.01 × 10^6^ Da) [20] and RNA 2 (1.4 kb), which encodes the viral coat protein (0.49 × 10^6^ Da) [21]. RNA 3 is a subgenomic transcript of the RNA1 segment which contains open reading frames for B1 and B2 non-structural proteins [22]. B1 inhibits apoptosis at the early stages of viral replication to release new viral particles from the infected cells [23], whereas B2 suppresses RNA silencing activity [22]. It is part of the genus *Betanodavirus* and is a member of the *Nodaviridae* family [12]. Based on a small variable sequence of RNA2 (T4 region), betanodaviruses have been classified into four genotypes, i.e., striped jack NNV (SJNNV), red-spotted grouper NNV (RGNNV), tiger puffer NNV (TPNNV), and barfin flounder NNV (BFNNV) [24]. Additionally, other genotypes have been proposed for NNV including, turbot NNV (TNV), Atlantic cod NNV (ACNNV), and Korean shellfish NNV (KSNNV); however, only TNV has been widely accepted as the fifth genotype [13]. In recent years, the sequencing of both genomic segments has proved the existence of natural reassortants between the RGNNV and SJNNV, with both SJNNV/RGNNV and RGNNV/SJNNV combinations [25,26]. Viruses belonging to different genotypes exhibit different host ranges, and reports from South Europe, the Atlantic coast, and the Mediterranean basin refer to SJNNV and RGNNV genotypes [27,28]. Currently, the RGNNV genotype is the most widely distributed and has the highest number of susceptible species in farmed and wild fish.

The present review aims to summarize and analyze the current knowledge of gene expression responses to nervous necrosis virus (NNV) infection in teleost fish species using published transcriptomic data (microarrays and RNA-seq). Four electronic databases (PubMed, Web of Science, SCOPUS, Google Scholar) were searched with appropriate keywords until 1 May 2021. More than 500 publications were identified across databases and manual searches. Following duplicate removal, title and abstract screening, and full-text evaluation, 24 articles were characterized as eligible for the present review based on selected criteria. The present review summarizes the current knowledge of the NNV challenge of different host species in different developmental stages and tissues, aiming to highlight the genes and the pathways that are most affected by the viral infection. Recognition of universal host reactions mechanisms will enable novel biomarkers assessment and will facilitate future studies design.

## 3. Results

### 3.1. Search Strategy and Outcomes Analysis

**Figure 1:**
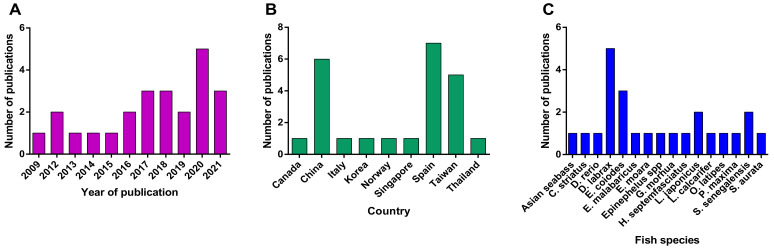
(**A**) Research articles by year of publication. (**B**) Countries of origin for research groups employed in transcriptome studies. (**C**) Fish species analyzed in NNV infection transcriptome studies.

Four sets of keywords were used to perform a complete search regarding transcriptomic analysis of betanodavirus infected fish hosts, as described in Section 5.1. For the term “transcriptome AND nervous necrosis virus,” 573 hits were identified across the four electronic databases. The term “transcriptome AND betanodavirus” resulted in 318 hits, the term “microarrays AND nervous necrosis virus” resulted in 198 hits, and the term “microarrays AND betanodavirus” resulted in 88 hits. After removing duplicated articles for each search term in at least two of the four databases, the remaining articles were tested with the eligibility criteria using titles, abstracts, and full texts. At the end of the screening procedure, the eligible articles for each term were as follows: “transcriptome AND nervous necrosis virus” referred in 20 articles, the term “transcriptome AND betanodavirus” was found in 22 articles, the term “microarrays AND nervous necrosis virus” resulted in 4 hits and the term “microarrays AND betanodavirus” resulted in 6 eligible hits. Finally, removing duplicated articles between the search terms resulted in 24 eligible articles. The article information regarding fish species analyzed organs, sampling time point, transcriptome methods, and the number of up- and downregulated DEGs are summarized in Table 1 and Figure 1.

The first publication regarding transcriptomic analysis of betanodavirus infected fish host utilizing microarrays was published in 2009 (Figure 1A). Interestingly, an increasing trend in transcriptome analysis in various fish species has been observed in the last few years, possibly due to the wider application of RNA-seq technology. Fish and Shellfish Immunology (12 articles) and the Journal of Fish Diseases (3 articles) are the top two journals with publications in the object. Each of the remaining articles has been published in Aquaculture Reports, Biology (MDPI), BMC Genomics, Frontiers in Immunology, Gene, Genes (MDPI), Molecular Immunology, Scientific Reports, and Veterinary Research. Three of these journals are directly associated with fish studies, while the others belong in genomics and immunology fields in general, suggesting that the transcriptional response to fish infections is a subject of general interest, a fact also observed by Caruffo et al. [52]. 

**Figure 2:**
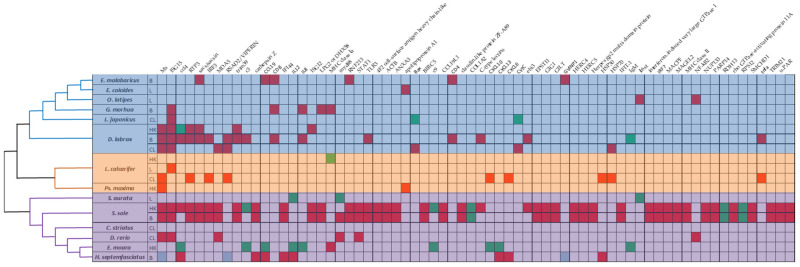
Diagram of fish and genes information of the articles incorporated in systematic review. (**Top**) genes which appear at least 2 times in different fish species/tissues (red, up-regulated genes; green, down-regulated genes; blue, de-regulated genes). (**Left**) phylogeny of the fish species, including different analyzed tissue information (B, brain; C, cell line; HK, head kidney; L, larvae). The tree is not drawn to scale.

Most studies on betanodavirus transcriptomics have been performed in countries that are heavily involved in the aquaculture industry (Figure 1B); therefore, the studied fish species belong to the most intensively farmed species of each region, e.g., *Dicentrarchus labrax* in Europe and *Epinephelus*spp. in Asia (Figure 1C and Figure 2). In detail, fourteen different fish species were investigated in the frame of transcriptome analysis following betanodavirus infection. A neighbor-joining cladogram of the fish species showed that they were grouped in three clusters and that the one formed by *E. septemfasciatus*, *E. moara*, *D. rerio*, *C. striata*, *S. senegalensis*, and *S. aurata* contained the highest number of de-regulated genes (Figure 2). 

The most frequently used betanodavirus genotype was the RGNNV genotype (15 articles) (Table S1), while a reassortant betanodavirus strain (RGNNV/SJNNV) was used in 3 studies. The host transcriptomic profiles after infection with betanodavirus were studied at various time points to assess acute and chronic/persistent effects of the infection (Table 1). Finally, the most analyzed tissues were the brain (*n* = 8) and head kidney (*n* = 8), since they are the target organ of betanodavirus and the major immune organ in fish, respectively.

### 3.2. Transcriptome Analysis Platform Information

The vast majority of the published studies have utilized the RNA-Seq technology (17 articles, 70.8%) provided by Illumina (San Diego, CA, USA) on platforms HiSeq 2000, Hiseq 2500, HighSeq 4000, and NextSeq 500 (Table S2). Microarrays were mostly used up to 2015 since that technology was more accessible at that time. In the last two years, an alternative platform for partial transcriptome analysis has been utilized by a research group from Spain based on custom-made high-performance OpenArray chip technology provided by Thermofisher (Waltham, MA, USA), significantly lowering the overall cost for partial transcriptome analysis. In most cases (17 out of 24, 70.8%), the extracted RNAs from fish reared/treated/infected with the same conditions were mixed (pooled) before analysis since this approach has a lower cost. However, sample pooling could compromise biological variability by hiding unique sample aspects. For that reason, the last few years’ studies have been performed in individual samples (Table S2). 

### 3.3. Bioinformatic Methods for Transciptomic Data Analysis

The RNA-seq bioinformatics analysis comprises three steps: i. raw reads quality assessment and processing, ii. clean reads assembly, and iii. quantitative and/or functional analysis [8]. Analysis platforms usually generate FASTQ-format files subjected to adapter sequences removal, reads trimming and discarding based on their quality, and sequence reads filtration based on contig coverage. The tools that were used for these purposes are summarized in Table S2. Overall, when RNA-seq is performed on non-model animals, problems associated with functional annotation and enrichment analysis arise. Transcripts sequences are usually searched via databases, such as NCBI nucleotide sequences (NT), NCBI non-redundant protein (NR), Clusters of Orthologous Groups (COGs), KEGG, gene ontology (GO), and InterPro annotation. However, the results from the analysis of aquaculture species may be limited to generalized conclusions since low gene alignment is achieved. Currently, only the KEGG database shows a relatively high number of aligned genes, which allows for enrichment analysis of aquaculture species [10].

Even though protein-coding mRNA is traditionally used for transcriptome analysis, other RNA species are also analyzed by RNA-seq based methodologies since they are known to be involved in post-transcriptional regulation of gene expression and may be linked to immune functions as well as to the suppression of diseases of the host [8]. Those RNA include different types of non-coding (nc) RNAs, such as miRNA, siRNA, piRNA, snoRNAs, snRNA, lncRNA, and circRNA. 

## 4. Discussion

The immune system provides fish with the ability to resist pathogenic agents by both innate and adaptive immunity. Fish immune organs are slightly different from those of other higher vertebrates. The most well-known immune organ in fish is the head kidney (HK), formed by two Y-shaped arms that spread underneath the gills. The head, kidney, and thymus are considered the primary lymphoid organs in the fish immune system. Especially the anterior or head kidney is the main site for both hematopoiesis and lymphopoiesis, having the highest concentration of developing B and T lymphocytes, macrophages, granulocytes, and antibody-secreting cells. Fish lack lymph nodes and bone marrow, however similar to the mammalian immune system, they have secondary lymphoid organs such as spleen, liver, and mucosal-associated lymphoid tissues (MALT), sub-categorized into three tissues, i.e., gut-associated lymphoid tissue (GALT), gill-associated lymphoid tissue (GiALT) and skin-associated lymphoid tissue (SALT) [4,8]. 

From a physiology point of view, when the virus crosses anatomical barriers and penetrates host cells, many events are triggered by host–virus interactions. When nodavirus infects a fish upon intramuscular injection (IM), it initially spreads from the injection site to head-kidney, following to nervous tissues and finally to the brain [49]. Viral entry is believed to occur through clathrin-mediated endocytosis, and interaction with different cell receptors has been proposed, as summarized in [13]. Viruses have evolved processes to prevent or delay attacks by host cells to override the host defense mechanisms, and they try to manipulate the host machinery to produce material for viral replication [53]. From the other side of the ‘battle’, the hosts’ innate immune system employs several strategies of antiviral defense to survive a virus attack. In general, viral nucleic acids are detected by host cells by pattern recognition receptors (PRRs) [54]. PRRs in fish include retinoic acid-inducible gene-I-like receptors (RIG1-like receptors, RLR), which include melanoma differentiation-associated gene 5 (MDA5) and laboratory of genetics and physiology 2 (LGP2), also known as DHX58 [54,55]. Viral genome recognition by RLR induces a signaling pathway involving the TANK-binding kinase 1 (TBK1), facilitating interferon regulatory factors IRF3 and IRF7 activation and its translocation into the nucleus for the induction of interferon IFN-I [53,54]. TBK1 is also activated by the TBK binding protein (TBKBP1). Moreover, RLR and myeloid differentiation primary response (MyD88)—dependent TLR signaling pathways mediate type I IFN induction in response to RNA virus infection through NF-κB signaling pathway [54]. Toll-like receptors (TLRs) are important for the recognition of diverse pathogen-associated molecular patterns (PAMPs), such as polysaccharides, LPS, peptidoglycans, bacterial DNA, ds and ss viral RNA [56]. At least 22 TLR types have been identified in various fish, whereas TLR18-20, TLR22-28, and soluble TLR5S are considered fish-specific TLRs [57]. TLRs 7, 8, and 9 recognize ssRNAs, and MyD88 is their adaptor molecule [55]. Immune responses against betanodavirus infections include expression of IRF3, interleukins (IL-1b, IL-8, IL-10), tumor necrosis factor (TNF-a), transforming growth factor (TGF-b), cyclooxygenase (COX-2), viperin, and immunoglobulins (IgM), as well as non-immune-related proteins such as galectin, sacsin, and heat shock protein 90 (HSP90) [31]. NNV is an intracellular pathogen that causes apoptosis in its host [36], and it is likely that both B cell and cytotoxic T-cells’ activities are needed to provide an effective adaptive response against the virus. Presentation of antigens through an intracellular route induces MHC-I restricted CD8^+^ responses (i.e., cytotoxic T lymphocytes, CTLs), e.g., live, attenuated virus or DNA vaccines, while extracellular delivery induces MHC-II restricted CD4^+^ responses (i.e., antibody responses), e.g., inactivated virus and recombinant proteins [58]. Antibodies activation is an important host immune response during NNV infections in teleosts since the produced antibodies can neutralize the virus, preventing it from causing damage [59].

The nervous necrosis virus shows clear neurotropism. In fact, virus replication in the susceptible fish species seems to be restricted to nerve tissue, preferentially the brain and retina [60]. NNV causes neurons destruction by infection, and the virus replication results in vacuolation of the fish brain. In addition, it has been suggested that the observed damage could be the result of the brain inflammatory response since inflammation constitutes a defense mechanism against pathogens, but it may also damage cells and tissues [61]. Brain and eyes have been characterized as immune-privileged organs [62]. Immune privilege represents a special microenvironment where the systemic immune responses to antigenic molecules are remarkably reduced. An advantage of the immune privilege to the organism is that the damage generated during a “normal” immune response is attenuated and the tissue is protected; however, the foreign antigen is not removed and becomes a Trojan horse, waiting for the moment when privilege is lost and overwhelming tissue damage occurs. Even though the immune-privileged organ concept suggests the existence of different conditions that help control the access of pathogens to the tissue, it also enables the exacerbation of inflammation [63]. Therefore, the brain response to NNV is of great interest. Likewise, host responses of larvae and juvenile fish have been intensively studied since disease outbreaks have been reported mainly in early developmental stages, and affected larval neural tissue has a greater extent of vacuolation and in different brain areas than adult fish [13]. It should not be overlooked that several aspects of NNV-host interactions have been discovered using fish cell lines [43]; therefore, transcriptome analysis has also been applied to most of them under various conditions.

To decipher all aspects of NNV host responses, the present review is focused on transcriptome analysis of the main immune organ, i.e., the fish head kidney and the virus target organ (brain). Studies of spleen, liver, larvae, and whole fish, as well as results from cell lines transcriptome analysis, are also reported.

### 4.1. Kidney

The first study on NNV-infected fish was performed by Park et al. in 2009, in turbot head kidney [29]. Since then, NNV effects on head kidney transcriptome have been studied in several other species, including Senegalese sole [41,49], grouper [44], European sea bass [5,6,44,45,46], and Asian sea bass [47]. The most important de-regulated differentially expressed genes in the fish kidney are summarized in Table 2. 

***Turbot (Psetta maxima):*** Gene expression of *Psetta maxima* (turbot) kidney tissue was studied using a cDNA microarray with 1920 genes over a 72 h period [29]. Most of these genes were up-regulated, including two interferon-induced genes, i.e., Mx and interferon-inducible protein 35 (IFI35), and saxitoxin binding protein (BiP) 1, serum lectin isoform 4, and serum-inducible protein kinase genes, which had increased expression levels in at least one time point. Ceruloplasmin (CP), kininogen I, haptoglobin, thrombin, and proteinase-activated receptor 3 involved in complement pathway and coagulation cascade were also significantly up-regulated. On the other hand, significantly down-regulated genes included F-box only protein 25, 5-aminolevulinate synthase, and phosphatidylinositol 4-kinase. Overall, among the two interferon-stimulated genes (ISGs) found in this study, the expression of IFI35 regulates host antiviral response. There is evidence that IFI35 is associated with N-myc interactor (NMI) to form a high molecular mass complex (HMMC) in response to IFN-α treatment [64]. IFI35 can be reduced in response to cells treated with IFN-α/γ [65,66], interacts with signal transducer and activator of transcription proteins (STATs), is involved in the apoptosis process [67], and targets RIG-I for proteasome degradation in mammalian cells, facilitating vesicular stomatitis virus replication [68]. In a fish cell line study, it was recently found that IFI35 could function as a positive factor for snakehead vesiculovirus (SHVV) replication by negative regulation of RIG-I-like receptor signaling pathway genes [69]. The genes classified within the stress and/or defense response category included CP and haptoglobin (acute-phase proteins), BiP, and a serum lectin. With the exception of CP, which has known roles in iron homeostasis and protection of cells against free radical damage, the role of these genes in physiological and immunological responses is not well understood [70]. Finally, nodavirus infection resulted in significant up-regulation of several genes classified within the metabolism category, such as apolipoprotein A1 (ApoA-I), which in addition to its role in the maintenance of cortisol homeostasis also has an anti-inflammatory role [71].

***Senegalese sole (Solea senegalensis):*** To determine the role of immune response in the pathogenesis of betanodaviruses Labella et al. [41] determined differences in the transcriptomic profile of Senegalese sole after infection with two viruses: a highly virulent wild type (wt) reassortant (wSs160.3) [25] and a mutant strain (mut), which causes a 40% reduction in sole mortality compared to the wild type (rSs160.03247+270) [72], by RNA-seq. Several de-regulated genes were identified in both head kidney and brain/eye tissue tested in the same study. Collagen type I alpha 2 chain (COL1A2), which is involved in an inflammatory response and signaling pathways, was the only gene significantly down-regulated. Up-regulated differentially expressed genes (DEGs) indicated a clear relationship with the innate immune response against viral infection, including genes coding PRRs (LPG2 or DHX58) and IFN-stimulated genes (ISG15, Mx, STAT1, HERC5, IFI44, IFIT-1, NUP133, TRIM21). Moreover, apoptosis and cell proliferation-related genes (MACPF, u-PAR, PARP14, EPSTI1, ISG12), antigen processing and presentation genes (RNF213, HERC4, MHC class II), signaling pathways-related genes (ANXA3, RTP3), inflammatory response (CCL19L1), and cytoskeleton-extracellular matrix related-genes (SMCHD1, ACTB) were also highly expressed. Induction of the IFN I pathway after infection with the wt isolate was evident by up-regulation of several interferon-induced proteins, i.e., IFIT-I, ISG15, IFI44, GIG1, interferon-induced very large GTPase 1, IRF3, IRF7, and Mx. CCL19, a CC chemokine that induces migration of head kidney leukocytes and increases host immune response, was found up-regulated after the infection with the high virulent virus. It should be noted that only the DHX58 gene was upregulated in the head kidney after infection with the wt isolate, whereas no deregulation of RIG-I or MDA5 was observed in any experimental group and TLR3 was slightly upregulated. Apart from the up-regulation of type I IFN (IFN I) genes exclusively in animals infected with the wt isolate, other genes related to antiviral response were up-regulated, including genes related to protein ubiquitination (MAGEL2), antigen processing and presentation (GILT), virus responsive genes (VGRs) (Herpes gp2 multi-domain protein, claudin-like protein ZF-A89), inflammatory response genes (4F2 cell-surface antigen heavy chain-like), immune effectors (cathepsin Z) and genes related to apoptosis (BIRC5; rho GTPase-activating protein 11A, v-ets erythroblastosis virus E26 oncogene homolog 1). COL1A2, RDH13, and RPS12 were down-regulated following infection with the mutant strain, whereas DEGs related to C-type lectins recognition and signaling mechanisms were up-regulated. 

The gene ontology (GO) classification assigns DEGs into three main categories, namely biological process (BP), cellular component (CC), and molecular function (MF) [73]. GO analysis revealed that in head kidney samples inoculated with the wild type virus, the most frequently detected ontologies were “proteolysis” (BP), “integral component of membrane” (CC), and “ATP binding” (MF). For the samples inoculated with the mutant strain, the most predominant ontologies were “proteolysis” (BP), “intermediate filament” (CC), and “structural molecule activity” (MF). Cysteine-, serine-, and metalloproteases were the main enzyme families detected. Cell apoptosis, identified as proteolytic cleavage of cellular proteins, included the participation of cathepsins, serine proteases, calpains, and metalloproteases. Based on the transcriptome analysis results, the authors suggested that the signal transduction mechanisms triggered after the initial energy-dependent recognition of the viral pathogen through the fish cellular membrane using Ca^2+^ and Zn^2+^ were completely different for the two strains. In fact, immune response and proteolysis were induced by the wild type isolate, whereas proteolysis and vasculogenesis inhibition were utilized by the mutant strain [41].

Senegalese sole immune responses after infection with NNV of different virulence (wt and mut) were assessed by the OpenArray^®^ platform (Thermofisher, Waltham, MA, USA), based on quantitative PCR, in a time-course manner (2 and 3 dpi) [49]. In accordance with Labella et al. [41] findings, the genes that were mostly induced after infection with the wt virus were those related to the IFN-I pathway (viral recognition, regulation of IFN-I, JAK-STAT cascade, and ISGs) and virus response genes (VRGs). The highly virulent isolate induced up-regulation of MDA5, LGP2, TLR3, and several ISGs, including ISG12, ISG15, GIG1, and IFI44. Interestingly, however, GIG1 and IFI44 transcription decreased over time. IFIT1 and Mx were up-regulated by the wt virus and down-regulated following infection with the mutant strain. Regarding VRGs, LITAF, SACS, and TRIM39 were up-regulated by both viruses. Also, one of the two RTP3 unigenes displayed the highest expression of all DEGs in the study [49]. Infection with both viruses caused down-regulation of LMAN1, an ER-Golgi intermediate compartment 53 kDa protein (also known as ERGIC-53) which is an intracellular receptor that facilitates glycoprotein transport; however, NNV has non-enveloped viral particles and do not possess glycoproteins, and therefore lman1 down-regulation seems unexpected [74]. The complement factors C3, C9, and CFHR3 (complement factor H-related protein 3 precursors) were down-regulated with the wt isolate, which could be related to an attempt of the virus to regulate the inflammatory response during infection. Interestingly, the expression of chemokines CCL4, CCK, and EBI3 recorded 2 dpi, but pro-inflammatory cytokines (IL-1b or TNFa) were not detected [49].

***Grouper (Epinephelus moara):*** Asymptomatic and diseased *Epinephelus moara* were subjected to transcriptome sequencing of their immune tissues (kidney, liver, and spleen) [44]. A high number of significant DEGs were identified in diseased *E. moara* and many immune related genes were down-regulated in diseased grouper, including antibodies (IgM, IgD, IgT/IgZ), cellular mediated cytotoxic molecules (perforin), interleukins (IL1, IL2, IL8, IL11, IL12, IL15), complements (C1, C3, C7, C8, C9), chemokines (CCL4, CXCL13, CXCL10, CCL3, CCL19, CXCL9, CXCL12, CCL20, CCL25), chemokine receptors (CCR9, CXCR4, CXCR3, CXR1) and adhesion related genes. The expression of class I major histocompatibility complex (MHC) was significantly higher in all tested immune tissues. GO analysis of kidney DEGs revealed several enriched terms, including tetrapyrrole binding, in kidney and liver tissues. The following genes displayed strong expression correlations: ITGA2, CXCR1, Lag3, LAMA5, Sox11-b, PTGER4, RC3H2, IgD, LAMB2, IgM, ITGA6, LATS2, SEMA4B, GPR21, and Prf1, implying that they play important roles together in NNV infection control. By KEGG pathway enrichment analysis, many DEGs were enriched in metabolism and immune related pathways, such as phagosome, neuroactive ligand-receptor interaction and Fc gamma R-mediated phagocytosis, cell adhesion molecules (CAMs), ECM-receptor interaction, focal adhesion, the intestinal immune network for IgA production, cytokine-cytokine receptor interaction, apoptosis, endocytosis, natural killer cell-mediated cytotoxicity, as well as PPAR, TLRs, RLRs, NOD-like receptor, Jak-STAT, p53, TGF-beta, NF-kB, B cell receptor, Fc epsilon RI and MAPK signaling pathways. In particular, DEGs annotated in most of the immune related pathways showed consistently decreased expression in diseased grouper [44]. The authors note that even though endoplasmic reticulum (ER) stress response was not significantly enriched, 18 DEGs in the kidney were annotated in this pathway per previous findings [30,42]. Summarizing the study findings, Wang et al. state that even though class I MHC mRNA was expressed in the immune tissues of the diseased grouper, a significantly lower expression of antigen process related genes was shown, suggesting that the CTLs cytotoxicity was weaker in them. This observation was further strengthened by perforin down-regulation [44]. Perforin is an important immune molecule involved in CTL cytotoxicity, which directly contributes to T-cell mediated death via apoptosis or necrosis by the permeabilizing target cell membrane and ensuing translocation of pro-apoptotic granzymes into the cytoplasm [75]. Moreover, many DEGs in phagosome and lysosome pathways were also down-regulated in diseased fish. Also, the expression of class II MHC was significantly lower in the kidney of diseased *E. moara* [44].

***European sea bass (Dicentrarchus labrax):*** The complete transcriptome response of European sea bass head kidney to nodavirus infection at 24 and 72 hpi was studied by Lama et al. The number of identified DEGs increased over time, and most of the up-regulated genes encoded enzymes involved in the final steps of the steroid hormone biosynthesis pathway, specifically related to the transformation of cholesterol into cortisol: mitochondrial cytochrome p450 11b (CYP11b1), mitochondrial steroidogenic acute regulatory protein (STAR), steroid 21-hydroxylase (CYP21A), cytochrome p450 17a1 (CYP17a1), 3 beta-hydroxysteroid dehydrogenase (3BHSD) and mitochondrial cholesterol side-chain cleavage enzyme (CYP11A1). None of these genes were significantly modulated 72 hpi. Surprisingly, no typical immune genes seemed to be significantly modulated at both sampling points [45]. The modest modulation of immune genes contrasted with the broad, intense changes in the expression of genes involved in the hypothalamic-pituitary-interrenal (HPI) axis. HPI axis is activated under stress conditions and culminates in cortisol secretion by interrenal cells [76]. The only genes that were differentially modulated in the head kidney were those encoding enzymes involved in synthesizing cortisol from cholesterol. Cortisol modulates the immune response by provoking immunosuppression, explaining the nearly total absence of an immune response at 24 hpi and down-regulation of immune-related genes (e.g., IgM). Cortisol synthesis involves the production of reactive oxygen species (ROS) due to the activity of the steroidogenic cytochrome P450 enzymes. However, at 72 hpi, the down-regulation of genes encoding enzymes implicated in ROS production, including ero1-like protein alpha (ERO1a), hypoxia up-regulated protein 1 (HYOU1), protein disulfide-isomerase a4 (PDIA4), and quinone oxidoreductase pig3 (QORX) was observed. GO enrichment analyses revealed that enriched biological processes were only obtained at 24 hpi, with the terms “oxidation–reduction process”, “glucocorticoid biosynthetic process”, and “sterol metabolic process” [45]. 

The OpenArray^®^ platform was also used to perform a comparative analysis of DEGs in European sea bass inoculated with nodaviruses of different virulence. The main genes modulated after viral infection were those involved in the IFN I system and VRGs, whereas modulation of apoptosis-related genes was not recorded. The few observed DEGs involved down-regulation of anti-inflammatory genes (CCL4 and CXCL14) in the low virulent strain infected group. The most induced genes after NNV infection with the wt virus were VRGs, particularly RTP3, SACS, and TRIM39 [6]. Since betanodaviruses have been proven to cause cell death via mitochondrial targeting, Sacsin could be counteracting the NNV-induced cellular apoptosis [77]. RTP3 plays a role in the Asian sea bass resistance against NNV, and the direct anti-NNV activity of grouper TRIM39 has been demonstrated in over-expression experiments [78,79]. In addition, the most virulent virus induced a more intense response than the low virulent virus. In this study, MxA, MxB, ISG15, ISG12, and IFI56 were up-regulated, whereas VLIG was hardly induced in HK [6].

The long noncoding RNAs (lncRNAs) represent a subset of ncRNAs of over 200 nucleotides in length and are transcribed in the same way as mRNA. LncRNAs regulate the expression of adjacent genes (cis-acting regulation) or genes located at other genomic locations, even on different chromosomes (trans-acting) [80]. LncRNAs have been demonstrated to be involved in immune system regulation in mammals and teleost fish [81]. In work by Pereiro et al. [46], lncRNAs were uniquely identified in the head kidney of NNV infected European sea bass. Numerous immune terms, mainly related to the production of cytokines, were enriched, including Toll-like receptor signaling, inflammation, leukocyte activation and proliferation, complement pathway, and phagocytosis. The up-regulated lncRNAs were more than the down-regulated at 24 hpi, but these differences were substantially reduced at 72 hpi. The coding genes flanking the differentially expressed lncRNAs were analyzed by GO. A large number of biological process terms directly involved in immunity were found to be enriched, including ‘negative regulation of mast cell activation’, ‘positive regulation of immunoglobulin production’, ‘positive regulation of B cell proliferation’, ‘antigen processing and presentation of exogenous peptide antigen via MHC class II’, and ‘neutrophil mediated immunity’. At a later time-point, this immune representation seemed to have disappeared, although the immune-related term ‘leukotriene production involved in inflammatory response’ remains enriched. In the head kidney, some biological process terms suggesting DNA damage and cell cycle arrest were also observed (‘signal transduction involved in G2 DNA damage checkpoint,’ signal transduction involved in mitotic DNA damage checkpoint’, ‘DNA damage induced protein phosphorylation’, and ‘cell cycle arrest’). The terms ‘positive regulation of endoplasmic reticulum stress-induced intrinsic apoptotic signaling pathway’ and ‘positive regulation of glutathione biosynthetic process’ were also significantly enriched. As mentioned above by Lama et al. [45], analysis of the coding genes differentially modulated in the sea bass head kidney after NNV infection revealed enrichment in the oxidation-reduction process, indicating that these could be mechanisms controlled by lncRNAs [46].

***Asian seabass (Lates calcarifer):*** Head kidney RNA from RGNNV-infected and uninfected control fish 2 and 15 dpi were sequenced and compared. Functional analysis revealed DEGs implicated in autophagy (beclin-1, inositol 1,4,5-trisphosphate receptor (IP3R)), superoxide production (cytochrome b-245 light chain (CYBA), C3a anaphylatoxin chemotactic receptor (C3aR), neutrophil cytosol factor 2-like (NCF2)), inflammation (leukocyte surface antigen CD53), antigen presentation (MHC I alpha chain), antiviral activity (cholesterol 25-hydroxylase (CH25H)) and other immune functions (oxysterol-binding protein 1 (OSBP1), dipeptidyl peptidase I (cathepsin C), interleukin-5 receptor subunit alpha (IL5Rα)-like and tyrosine-protein kinase (BTK)) [47]. In more detail, among the up-regulated genes, OSBP1 is a member of the lipid transfer proteins family (LTPs) [82], beclin-1 is a key regulator of autophagy and membrane trafficking [83], and IP3R is another important player in autophagy [47]. CYBA encodes a light/alpha subunit that is a membrane-bound phagocyte NADPH oxidase, part of a microbial oxidase defense system [84], and cathepsin C is a lysosomal cysteine protease that plays a role in intracellular protein turnover [85]. IL5Rα or cluster of differentiation 125 (CD125) that regulates growth and activity of several leukocytes in mammals [86] was also highly abundant. Collectively, CYBA, C3aR, and NCF2-like transcripts up-regulation indicate that the virus induces oxidative stress [87]. On the other hand, the down-regulated genes include leukocyte surface antigen CD53, MHC class I alpha chain, and BTK. Finally, CH25H, an interferon-stimulated gene converting cholesterol to 25-hydroxycholesterol (25HC), was down-regulated. “Cellular process”, “binding”, and “cellular anatomical entity” was the most abundant subcategory in BP, MF, and CC GO terms, respectively. GO enrichment analysis further identified tubulin/microtubule (MTUB) binding and MTUB cytoskeleton-associated proteins suggesting RGNNV affects MTUB behavior and function in a pro-viral manner [47]. 

Overall, Angsujinda et al. [47] suggest that at 2 dpi, RGNNV subverts the host defense mechanism and promotes its RNA replication through activation of autophagic regulators. Moreover, the virus utilizes the lipid transfer proteins to support its cellular replication and interferes with the host cell’s innate antiviral response by down-regulation of interleukin enhancer binding factor 3 (ILF3/NF90) that possibly interacts with and inhibits viral RNA. The lower expression of CD53 and signal transducer and activator of transcription 5B-like (STAT5b) further reflect the viral manipulation of the host response. At a later stage of infection, the profound effect on host immunity was obvious as subunits of NADPH oxidase (CYBA, NCF2) required for pathogen clearance by phagocytes were enhanced. In addition, hyper-responsiveness to the infection and enhanced anti-RGNNV activity were suggested by the lower expression of suppressor of cytokine signaling 1 (SOCS1) and BTK. Decrease of type I IFN-induced CH25H and the limited activation of adaptive immunity through antigen presentation suggests the muting of the host response by a virus at this late time point [47].

### 4.2. Spleen

***Grouper (Epinephelus moara):*** Spleen analysis in diseased *E. moara* revealed similar patterns with kidney: spleen IgM and perforin 1 genes were down-regulated whereas HLA-A, MR1, C3, lysozyme, hepcidin, and CCL19 were up-regulated [44]. In the spleen, the enriched GO terms contained peptidase related pathways, potassium channel activity, and integral membrane components, whereas KEGG pathway analysis showed significantly enriched CAMs, ECM-receptor interaction, phagosome, focal adhesion, neuroactive ligand-receptor interaction, and regulation of actin cytoskeleton. In addition, DEGs belonged in the pathways of cytokine-cytokine receptor interaction, peroxisome, endocytosis, the intestinal immune network for IgA production, Jak-STAT signaling, p53 signaling, apoptosis, Toll-like receptor signaling, and MAPK signaling. Overall, antimicrobial peptides (i.e., hepcidin, lysozyme) and important innate immune molecules (c3, CCL19) were detected in high levels in the spleen of diseased grouper. High expressions of acute-phase protein serum amyloid A (SAA) and CD28 were also detected. SAA has been shown to possess many functional properties, including chemoattraction and opsonization of Gram-negative bacteria, which facilitated phagocytosis by macrophages, while CD28 plays a crucial role in maintaining regulatory T cell (Treg) pool size through promoting their development and proliferation.

### 4.3. Liver

***Grouper (Epinephelus moara):*** Liver analysis in diseased *E. moara* revealed) also indicated that MR1, C3, lysozyme, hepcidin, and CCL19 were up-regulated [44]. Liver DEGs were enriched in tetrapyrrole binding (as mentioned above in the kidney) and transport and localization-related pathways. KEGG analysis of liver DEGs indicated enrichment in the phagosome, antigen processing and presentation, CAMs, cytokine-cytokine receptor interaction, as well as PPAR and p53 signaling pathway. 

### 4.4. Brain

Up to date, 8 studies have been focused on the NNV target tissue transcriptomic profiling to elucidate pathways involved in brain pathogenesis. The studied fish species include Atlantic cod [32], Senegalese sole [41,49], grouper [38,42] and the European sea bass [6,45,46]. All referenced de-regulated DEGs in analyzed brains are summarized in Table 3.

***Atlantic cod (Gadus morhua):*** The transcriptomic changes in Atlantic cod brain 5, 11, and 25 dpi were analyzed by microarray. The majority of up-regulated genes were immune-associated and remained stable until 25 dpi. A strong activation of genes related to B and T cells’ pathogen recognition receptors, TLR and NLR, was reported. Specifically, TLR-like proteins, RNA helicase DHX58, and novel Ig-like/immune-type receptors (NILT and NITR) were strongly up-regulated. The rapid recruitment of B and T lymphocytes to the NNV infected brain was also supported by the fact that genes related to adaptive immune response, such as IFN gamma and immunoglobulin, were induced. This hypothesis was strengthened by the simultaneous up-regulation of CD3, CD8, TCR genes, and other T cell-specific regulators, namely RhoG1, ITK, FYB, LCK, and effectors like granzyme [32]. Furthermore, Krasnov et al. showed that an acute inflammation in the NNV infected brain was activated by the prominent induction of CC chemokines type-3 gene with simultaneous up-regulation of all eight IL-8 genes encoded in the Atlantic cod’s genome [88] and the induction of IL17a. TNFa and NF-kappaB pathway-related genes and their immune effectors were also induced. A strong communication between cells in the infected brain tissue was observed as CAMs, focal adhesion, and leucocyte transendothelial migration was activated, along with the induction of cellular and humoral factors. The enrichment of two main domains in the encoded genes during infection was highlighted during analysis, i.e., NACHT domain which possesses NTPase activity [89] and is found in NLRs that recognize microbial and viral components [90], and genes encoding PRY-SPRY domains present in multiple proteins of innate immunity [91], suggesting that proteins containing such domains may act as pathogen sensors and promote the establishment of antiviral responses in fish. However, the most prominent response to NNV infection in the Atlantic cod’s brain was attributed to genes involved in immediate innate responses to viruses. These included VRGs, members of the JAK-STAT pathway, and other signal transducers like IRFs and specialized effectors such as GTPases, ISG15, and ubiquitin related proteins. Genes related to antigen presentation, such as components of MHC I complex-B2M and multiple MHC Ia antigens, tapasins, and proteasome components, were also activated during the infection [32]. 

***Senegalese sole (Solea senegalensis):*** Interestingly, similar pathways were shown to be activated in a betanodavirus infected Senegalese sole’s brain 2 dpi with wild type NNV isolate after analysis with the OpenArray platform [49]. In fact, a high number of genes relevant to viral infection and its severity, i.e., genes related to immune response and proteolysis were differentially expressed. Genes that are associated with viral recognition, regulation of the IFN-I pathway, as well as genes related to JAK-STAT cascade, ISGs, and VRGs were found to be highly induced upon infection. However, it was shown that the virulent isolate mediated immune response induced interferon I exacerbation in the sole brain, which resulted in the disease’s severity progression instead of having a protecting effect. Instead, apoptosis, suppression of cell proliferation and inflammation-derived immunopathology were promoted [92]. In more detail, MDA5, one of the RIG1-like receptors, was only expressed in the sole brain, 2 dpi with the highly virulent isolate, which could result in Mx accumulation [93]. On the other hand, sole infection with the mutant moderately virulent isolate resulted in up-regulation of MyD88 and TANK-binding kinase 1 binding protein (TBKBP1, also known as SINTAD) genes in the nervous tissues of the fish, suggesting that the viral recognition happens through TLRs (TLR7, 8 or 9), adaptor (MyD88), and the TBKBP1 [49]. The overexpression of MyD88 has previously been reported to induce IFN I through the NF-κB signaling pathway [94]. Interferon stimulated genes’ up-regulation was observed in fish nervous tissues infected with the wt NNV isolate (i.e., ISG15, IFIT1, Mx, GIG1, IFI44). Taking into consideration that IRFs and several ISGs are known to be pro-apoptotic [95], the expression of IRF3, IRF7, IFI44, and PKR in the sole’s infected brain could lead to apoptosis of the infected cells [49]. That hypothesis is also partially anticipated after stimulation of the IFNs and the exacerbated IFN-I response [92]. The authors reported induction of genes involved in IFN-I-inducible enzymes response related to protein ubiquitination (E3-ligases HERC4 and HERC5) involved in ISGylation, an antiviral activity held by the ubiquitin-like protein ISG15 [96]. Regarding VRGs, a higher expression of sacs and trim39 genes was observed in the tissue 2 dpi. Sacs belonging to sacsin regulators of heat shock protein 70 (HSP70) chaperon machinery to promote betanodavirus-induced stress while trim39 gene has an important role in viral and bacterial infections [49].

The study by Labella et al. [49] was in accordance with a previous study reporting the transcriptomic changes in the Senegalese sole brain with a wild-type (wSs160.03) and a mutant isolate (rSs160.03247+270) [41]. It was shown that the predominant biological processes that were influenced by infection with the wild-type isolate were immune response and proteolysis, while the mutant isolate mainly inhibited vasculogenesis in nervous tissue. In more detail, infection with the wild type high virulent NNV isolate presented an induction of immune genes related to: (a) PRRs (DHX58), (b) mediators of IFN-I expression pathway (IRF3, IRF7), and (c) IFN-stimulated genes, apoptosis, and antigen presentation (ISG15, Mx, PKR, GIG1, ISG12, IFI44, IFIT-1, STAT1, HERC5, NUP133, TRIM21, MACPF, u-PAR, PARP14, EPSTI1, RNF213, HERC4 and MHC class II genes). Moreover, genes related to signaling pathways ANXA3 and RTP3, inflammatory response (chemokines such as CCL19L1), cytoskeleton, and extracellular matrix (SMCHD1 and ACTB) were also induced, while COL1A2, a gene involved in both inflammatory response and signaling pathways, was downregulated. Regarding the mutant virus strain, COL1A2 was also downregulated in the sole brain along with RDH13 and RPS12, indicating that signaling pathways (JNK, TLRs, protein ubiquitination, G-protein) and inflammatory response were down-regulated in eye/brain samples [41].

***Grouper (Hyporthodus septemfasciatus):*** Transcriptomic analysis of NNV-infected sevenband grouper indicated that a highly differentiated expression of immune genes was observed [38]. Several cytokines (CCL2, CCL3, CCL19, CCL4, CXCL13, CXCL6, CXCL8, CXCL9, IL12a, IL12b, and IL18b) were highly up-regulated. Although different cytokines were induced, in accordance with observations of Senegalese sole’s and Atlantic cod’s infected brains, the authors reported for the first time that the CCL2 gene was one of the most highly expressed genes in the grouper brain. CCL2 is already known to have a role in the neuro-inflammatory response in the nervous system, acting as a pro-inflammatory chemokine during different viral infections [97,98,99,100,101,102,103]. Lysosomal cysteine enzymes, important for cellular homeostasis and innate immune response (cathepsin family, subtypes L, H, K, O, S, and Z), were also induced in the infected grouper brain. Cathepsin L and cathepsin S, known to be involved in regulating antigen presentation and degradation, apoptosis, inflammation, and hormone processing regulation, were the most highly expressed lysosomal cysteine enzymes following NNV infection. Other DEGs reported in the study include C-type lectins (CLEC4M, CLEC10A) and their receptor (CD209), galectins (LGALS9, LGALS3), fucolectin (FUCL4), mannose-binding lectin (MBL), antiviral and antibacterial proteins (radical S-adenosyl methionin domain-containing protein 2 (RSAD2 or viperin), NK-Lysin,) IFN-I effectors or IFN-I induced proteins (Mx, IFI44, IFIT5, GVINP1, EIF2Ak2, IFIH1). Interestingly, the above-mentioned IFI44 gen induced in the Senegalese sole’s brain upon NNV infection was also one of the most expressed ISGs in the grouper’s brain 2 dpi [41]. Moreover, caspase, cathepsins, IRF, viperin, and TRIM genes’ expression were also observed in the Atlantic cod brain during NNV infection [32]. It is worth mentioning that amongst the heat shock proteins family (HSPs), only the HSP30 gene was significantly upregulated in the NNV-infected grouper’s brain [38]. 

***Grouper (Epinephelus malabaricus):*** A second study focused on the transcriptomic analysis during persistent NNV infection in grouper’s (*Epinephelus malabaricus*) brain rather than the acute phase of the disease [42]. As expected, the immune response, especially the interferon-induced response, as well as the surface receptor expression, were highly activated during this phase of infection. Intriguingly, the authors showed that negative regulatory factors of T-cell receptor signaling, i.e., PD-L1 and LAG3, were triggered in the persistent state of infection in the grouper’s brain, resulting in the exhaustion of T-cell response, a common phenomenon in chronic and persistent inflammatory infections. However, simultaneously, T-cell surface proteins such as CD3E, CD4, and CD8A, also considered markers of chronic and persistent infections, were up-regulated. The RNF213 gene showed high expression levels, suggesting that it plays a role in the T-cell differentiation as its abnormality has been associated with defective differentiation of regulatory T-cells [104]. Similar to the Senegalese sole, CCL19, which induces dendritic cell maturation and T-cell proliferation, was one of the most up-regulated genes in the grouper’s brain. The overexpression of the CCL19 gene is reasonable as it might play a fundamental role in the migration of the activated T-cell effectors at the damaged tissue of the central nervous system during NNV infection. Along with CCL19, the exonuclease’s TREX gene, known to participate in the antiviral response, was highly triggered. Sacsin, a gene overexpressed in the brain of both Atlantic cod [32] and Senegalese sole when infected [49], was also up-regulated in the nodavirus-infected grouper’s brain, indicating that it is possibly expressed in the infected fish brain to prevent the stress and neurodegenerative effects of the disease and to prevent protein misfolding and aggregation when it cooperates with Hsp70 and Hsp90. Another mutual up-regulated gene between the Atlantic cod and the grouper was the ZNFX1, which is a potent regulator of inflammatory response’s duration by acting as transcriptional repressors. Finally, the IFN-inducible GTPase GVINP1, a factor that has a role in the pathogen-invading response of the cells, seems to be highly expressed in the grouper’s brain and is thought to possess an important role in the anti-NNV defense during persistent infection. The authors also demonstrated specific genes that were down-regulated in the brain during persistent NNV infection. Those genes were mostly related to neuronal, synaptic function, DNA damage response, telomere maintenance, or negative regulation of RIG-I and type I IFN signaling (NPTN immunoglobulin, SF3B1, EWSR1, NLRX1, GNG3, and MGEA5) [42].

***European sea bass (Dicentrarchus labrax):*** The complete transcriptome response of European sea bass to NNV infection in early time-points (24 and 72 hpi) was evaluated by Lama et al. [45]. The data revealed low modulation of immune-related genes in the infected fish brain 24 hpi since only 10 genes were de-regulated. C-type lectin, leukocyte cell-derived chemotaxin-2 (LECT2), and the NKTR receptor of natural killer cells were overexpressed, whereas genes including immunoglobulin mu chain C region secreted form (IgHM), stimulator of interferon genes protein (STING), and NLR family CARD domain containing 3 (NLRC3) were down-regulated. These findings imply that the latter responses were probably postponed to allow the organism to prioritize its stress mechanisms. At 72 hpi, immune genes’ overexpression was observed in the brain since the stress insisted. More specifically, pentraxin 4 (PTX4), viperin, complement component c3 (C3), and complement c1q tumor necrosis factor-related protein 1 (C1QT1) were up-regulated. IFN-I pathway-associated genes were absent from the transcriptomic data, a fact that could be attributed to the mediated high-stress response at the early points of infection in an immune privileged organ. Interestingly, high numbers of genes related to hormones involved in the HPI axis were modulated during NNV infection in the brain. Growth-related genes, i.e., prolactin (PRL), somatolactin (SL), somatotropin (SOMA) or growth hormone (GH), gonadotropin (GLHA), thyrotropin (TSH), and pro-opiomelanocortin (POMC) were upregulated 72 hpi, even though PRL, SL, and SOMA were down-regulated 24 hpi. POMC is known to encode the precursor protein of adrenocorticotropic hormone (ACTH), which in its turn induces cortisol, the main active corticosteroid in fish [105]. Although POMC was up-regulated at 72 hpi, no modulation of genes involved in cortisol synthesis was observed. It should be noted that in the present study, the stress response was activated 24 hpi but attenuated at 72 hpi. Neurotransmitter receptors, LECT2, and the glutamate receptor ionotropic NMDE2 gene switched from up-regulation at 24 hpi to down-regulation at 72 hpi. At the same time, the serotonin receptor 5-hydroxytryptamine receptor 3E (5HT3E) was up-regulated in the highest degree at 24 hpi. Gamma-aminobutyric acid (GABA) receptor was mostly up-regulated 72 hpi; however, the adrenergic receptors which regulate the HPI axis were down-regulated. Furthermore, there was a high representation of genes related to calcium homeostasis and transport with overexpression of calcium-related genes at 24 hpi. This fact, in combination with the up-regulation of genes involved in ROS production (i.e., ecto-NOX disulfide-thiol exchanger 1), was linked to excitotoxicity. In general, neurotransmitters can cause excitotoxicity by increasing the massive influx of calcium ions into cells, a fact that could result in damaging the neuronal tissue and promoting an immunosuppressive status, favoring the NNV infection. However, most of these genes, including nitric oxide synthase gene NOS1, were down-regulated at 72 hpi, probably as a result of the effort of the immune system to compensate for the damage that occurred in earlier time-points of the infection. As a consequence, the host seems to initiate a response to the NNV infection upon 72 hpi [45]. 

In a related study, analysis of the lncRNAs indicated their pivotal role in European sea bass response to NNV infection [46]. In this study, lncRNAs analysis highlighted the modulation of genes associated with immune response, viral infectivity, and stress response and of genes directly related to the nervous system, as expected. To that end, only one lncRNA seems to regulate an immune-related gene 24 hpi to produce antibodies (‘positive regulation of isotype switching to IgA isotypes’). A flanking lncRNA to C3 was also up-regulated 24 hpi, in accordance with C3 gene overexpression. On the other hand, NK-tumor recognition protein (NKTRP) was correlated to three up-regulated lncRNAs while three up-regulated and one inversely-correlated lncRNAs were found in the vicinity of cerebellin 1 (CBLN1). Moreover, upregulation of a lncRNA near beta-1,4-galactosyltransferase 1 (B4GALT1) gene was correlated to its overexpression in the brain. As far as calcium homeostasis was concerned, sarcoplasmic endoplasmic reticulum calcium ATPase 1-like (ATP2A1 or SERCA1) and g-protein signaling 6-like (RGS6) were modulated during NNV infection [45] and also had a putative correlation with the expression of neighboring lncRNAs. Finally, lncRNAs regulating genes that induce DNA damage response were also reported due to excitotoxicity [46].

Finally, Moreno et al. utilized the OpenArray^®^ platform to quantify the transcriptional levels of 56 immuno-related genes in the NNV-infected sea bass brain. As before, two virus strains were used: a highly virulent wild type isolate and a mutated strain (Mut247+270Dl956) with lower virulence [6]. The resulting data were comparable to those obtained with the same methodology from infected Senegalese sole’s brain [49]. As expected, genes responses were more abundant when the sea bass was infected with the wt isolate, resulting in DEGs related to the IFN I pathway (ISGs, IRFs, PRRs, adaptor proteins, and negative regulators of the IFN-I system), inflammatory response, cell-mediated response, and apoptosis. Infection with the mutant strain resulted in a delayed response of the IFN-I pathway in early and transitory inflammation and cell-mediated responses. The authors pointed out that an early and strong response of the IFN-I pathway (in the case of the virulent isolate) may induce damage in the brain, enhancing the viral pathogenesis and spreading instead of protecting the tissue from the virus. Several studies suggest that a strong immune response and the activation of pro-inflammatory mechanisms may induce severe damage in the targeted tissue, but if there is a complete absence of such response, it would probably result in a persisting and highly spreading infection. Thus a strategic balance of the immune response is essential during NNV infection [46,49]. When fish were infected with the wt isolate, DEGs increase seemed to reach a maximum at 5 dpi while infection with the mutant strain showed a higher increase in the target organ from 24 hpi, suggesting that animals that were infected with a less virulent isolate express immune molecules faster to control the viral infection. The VRGs RTP3, SACS, and TRIM39 were the most induced DEGs indicating their antiviral role. Sacsin, which seems to be overexpressed in the infected brain of all studied fish (i.e., Atlantic cod, Senegalese sole, grouper, and European sea bass), has been suggested to regulate mitochondrial dynamics, thus protecting against NNV by inhibiting apoptosis caused via mitochondrial targeting. Cathepsin was the only pro-apoptotic gene that was induced in the NNV-infected sea bass brain in accordance with data acquired from sevenband grouper [38]. Manifestation of the IFN-I pathway in infected sea bass brain appeared through up-regulation of TLRs (TLR3, TLR8, TLR21), IRF3, and of almost all ISGs, with ISG15 and MxA most predominant. TLR3 seems to have a lesser role during the NNV infection, a fact that has also been reported for Senegalese sole [41]. Transcription of inflammatory genes, however, seems to be differently induced by the wild type isolate and the mutant strain. TNF-a was equally triggered in both cases, however, the pro-inflammatory genes IL-8 and IL-1b transcribed upon TNF-a induction seem to have a higher expression following infection with the mutant isolate. The anti-inflammatory genes CCL4 and EBI3 were induced earlier (5 dpi) upon infection with the wt isolate. Regarding the cell-mediated response related genes, NCCRP-1, TCR-γ, CD4, and CD8 were up-regulated in the sea bass brain with higher levels of NCCRP1 and TCR-γ upon infection with the mutant isolate at 3 dpi, which could be related to the up-regulation of IL-1b. It is worth mentioning that IL-1b is known to enhance neutrophils’, macrophages’, and lymphocytes’ trafficking. Even though TCR-γ is a co-receptor of CD8 and CD4 in cytotoxic and helper lymphocytes, it seems to be up-regulated upon infection with the mutant low-virulent strain (especially 3 dpi) without simultaneous up-regulation of CD8 and CD4, suggesting that T-cells are not present in the target tissue at that time point [6].

### 4.5. Larvae

While host response to NNV has been extensively studied in adult fish, little attention has been devoted to early life stages, generally the most sensitive ones [50]. At present, 3 transcriptome studies have been focused on the larvae developmental stage of orange spotted grouper [31,48] and gilthead sea bream [50]. Moreover, a transcriptomic analysis has been performed in a small-sized whole fish (medaka) [33]. The most important de-regulated DEGs in these tissue categories are summarized in Table 4.

***Grouper (Epinephelus coioides):*** Morbid and healthy larvae of *Epinephelus coioides* with various viral content levels (high, medium, and low) were analyzed by microarrays, and the results were validated by gene-specific qPCR [106]. Overall, the trend of 14 up-regulated genes was negatively correlated with the larvae viral content. Among these genes, H-like myosin binding protein (MyBP-H), myosin light chain 2 (MLC2), and the embryonic isoform of fast/white muscle troponin T (TnT) were up-regulated in all three virus-containing larvae. In contrast, adenylate kinase 1–2 (ADK1-2), myosin light chain 3 (MLC3), tropomyosin (TM), and two types of parvalbumin (PV1 and PV2) were significantly induced only in the high virus level larvae. The NNV-infected larvae displayed unnatural swimming patterns, which might have been due to functional deficiencies in some movement- or nerve-related genes. Indeed, MLC2, MLC3, Mybp-H, TM, TnT, and PV are plentiful in skeletal muscle cells, implying involvement in muscle contraction, and ADK is a phosphotransferase enzyme that participates in adenine nucleotide interconversion [106]. PV has a helix-loop-helix structure that can specifically bind calcium ions, and its distribution includes skeletal muscles, the brain, and the retinas. In addition to contributing to the acceleration of muscle movements, PV is also implicated in the CNS. Four down-regulated genes were positively correlated with the viral content of larvae, including ApoA-I and trypsinogen. ApoA-I is the major protein component of high-density lipoprotein in plasma and is implicated in the efflux of cellular cholesterol from tissues to the liver, while trypsinogen is the precursor of the pancreatic enzyme trypsin, a serine protease found in the digestive system. Abnormal trypsinogen activation might cause cell apoptosis. After nodavirus infection, the virus might induce expression of ApoA-I and trypsinogen to interfere with host self-defense systems to create a suitable environment for virus propagation [31].

Ge et al. [48] used RNA-seq and label-free, quantitative mass spectrometry to investigate the dynamic multi-omic changes of the orange-spotted grouper larvae in the infected/dead and infected/survival (persistent) stage. Overlapping genes between the transcriptome and proteome point to an elevated expression of collagens in the persistent stage. Enhancement of lipid metabolism in the NNV-infected/dead groupers was also found, suggesting that NNV infection alters the homeostasis of metabolism. Even though there were significant numbers of differentially expressed genes, overlapping genes between the transcriptomes and proteomes were limited, and most of them were related to immune responses. In the infected/dead larvae, three of the up-regulated genes might play a role in the immune response: tubulin beta-1 chain, PAST-1, and serine/threonine-protein phosphatase 5 or Aurora kinase B (AURKB). Microtubules which contain β-tubulin are essential for the formation of macroplatelets and hyper-aggregation of platelets after stimulation, PAST-1 plays a role in the endocytic recycling regulatory proteins in T cell receptor (TCR)-mediated T cell functions, and AURKB is a central regulator of chromosome segregation and cytokinesis [107,108,109]. On the other hand, the down-regulated immune-related genes in the infected/dead vs. control group are complement C5, a part of the innate immune system that plays an important role in inflammation, host homeostasis, and defense against pathogens, and retinol-binding protein 4-A like, which is an adipocyte-derived protein, and its elevation causes adipose tissue inflammation by activating innate immunity [110,111]. Two genes were down-regulated in the infected/survival larvae compared to the control group: HEAT repeat-containing protein 1, which was reported to induce functional cytotoxic T lymphocytes in glioma patients importin-9, which is a specific, and negative, post-transcriptional regulator of IFN-ε expression [112,113]. 

The biggest group of overlapping genes was the up-regulated genes in infected/survival larvae compared to control [48]. Among the genes related to immune responses, the authors reported integrin alpha-V isoform X1 and integrin beta-4, which mediate macrophage chemotaxis and infiltration, and mark a rare population of cancer stem cells that drives tumor propagation, respectively. Other up-regulated genes include anoctamin-10 (ANO10), which encoded Cl^−^ channels and phospholipid scramblases supporting the migration of macrophages, and chymotrypsinogen, a nuclear receptor for epithelial renewal and homeostasis against TNFα-damage and T-cell-mediated immune responses [114,115]. The adhesion molecules claudin-15-like, afadin isoform X8, and cadherin-17 were also highly expressed. Adhesion molecules regulate the mechanical interactions between cells, playing a central role in leukocyte extravasation and immune response [116]. Collagen type I alpha 3 chain, collagen alpha-1(XVIII) chain isoform X2, and collagen alpha-2(V) chain were also up-regulated. Collagens represent the most abundant molecules in the dense extracellular matrix and modulate centrally cellular functions and physiological processes. Collagens can modify immune reactions mechanically by sequestering or displaying growth factors and by interacting with immune cells. Collagen-derived peptides can also modulate T-cell differentiation and suppress allergic responses [117,118]. Another gene, procollagen-lysine, 2-oxoglutarate 5-dioxygenase 1 isoform X2, was also related to collagen synthesis since it catalyzes lysyl hydroxylation, an essential step for collagen cross-link and deposition. Overall, the authors conclude that grouper larvae persistent to NNV infection up-regulated genes related to cytoskeleton, adhesion molecules, and collagen synthesis, which might suppress the acute and lethal immune responses upon NNV infection and maintain the larvae in the persistent stage [48].

***Gilthead sea bream (Sparus aurata):*** The transcriptomes of gilthead sea bream larvae experimentally infected with an RGNNV/SJNNV strain were analyzed at four time points (6–48 hpi). Up-regulated DEGs at the early time points belonged to heat shock proteins (HSPs) family (HSPA5 or glucose-regulated protein 78—GRP78, HSPA9, HSC70, TRAP1, UNC45b, FKBP4, PTGES3A) and to endocytosis pathway (RAB10, RAB11A, AP2a1, CHMP2a), while many immune genes were down-regulated (MyD88, IRF5, PIK3R1, STAT3, JAK1, IL12b, IL6st). In general, HSPs are crucial for cell homeostasis and prevention of cellular stress negative effects. They are induced during NNV infection and have a double role by contributing to the antigen presentation process and enabling the assembly of viral replication complexes (VRCs) on intracellular membranes [35,43]. The prolyl isomerase Fkbp4 gene has a role in stabilizing the viral replication complexes, while Hspa5 is a key gene involved in relieving cellular stress by triggering the “unfolding protein response pathway” and is also known to interact with NNV capsid and its RNA-dependent RNA polymerase [30]. Many PEX genes (PEX3, PEX26, PEX11), which are essential for peroxisome’s maturation and growth [119], and peroxisomal enzymes involved in lipid metabolism (EPHX2, ACOT8) or antioxidant systems (GSTK1) were intensely down-regulated in the initial stages of the infection while the PEX genes PEX5la and PEX26 were upregulated 48 hpi. Among the down-regulated immune genes, two were particularly relevant to NNV infection, i.e., MyD88 and IRF5. MyD88 is activated by nearly all TLR proteins, and it is essential for the inflammatory response via regulation of inflammatory cytokines, whereas IRF5 is a transcription factor expressed at high levels in fish immune organs and has an important role in immune defense against intracellular pathogens. Hence, their down-regulation at the initial stage of the infection is unexpected since they are two key proteins of the TLR pathway and might play an important role favoring the NNV evasion from an immune response. Mapping of gene expression changes at different time points by KEGG pathway analysis indicated that key components of the autophagy pathway (either controlling phagophore formation or its fusion with lysosome) were down-regulated early after infection (e.g., LC3, ATG9L, STX17, VAMP8), while others were up-regulated at 48 h (e.g., ATG4, ATG16, BNIP3, DPCP1).

The gene set enrichment analysis (GSEA) revealed coordinated shifts in gene expression across multiple genes within the same biological pathway/process, including pathways/processes of peroxisomes formation, which are important anti-viral components and essential for nervous system homeostasis, platelets/neutrophils degranulation, and the autophagy pathway, all down-regulated at early time points. All such processes are tightly interconnected with cellular activities that cooperate to maintain cytoplasm homeostasis. Interestingly, the autophagy process was up-regulated at a later time point (48 hpi). The NF-kB activation pathway and genes belonging to “Class I MHC mediated antigen processing & presentation” were also up-regulated. SOCS1a, WDR55, ERAP2, HECTD2, FBXl18, NCF2, and CUL1a were some of the most up-regulated genes of this enrichment core. SOCS1a is a negative regulator of cytokine and growth factor receptor signaling and is crucial in attenuating IFN signaling. ERAP2 and its homologous ERAP1 plays an essential role in the generation of peptides that serve as ligands for MHC class I (MHC-1) molecules, and NCF2 encodes a subunit of the NADPH oxidase enzyme complex, which regulates dendritic cell (DC) cross-presentation by MHC-1 molecules through regulation of the phagosomal microenvironment. Over representation analysis (ORA) revealed biological processes/functions enriched in “heat shock protein binding”, “unfolded protein binding”, and “chaperone-mediated binding” (up-regulated) while “visual perception” and “sensory perception of light stimulus” were found enriched in down-regulated DEGs. Eye formation and development is a key developmental process in early fish larval stages. Such development might be impaired by NNV, or eye function might be inhibited due to the damages caused by viral replication [50].

***Medaka (Oryzias latipes)*:** Wang et al. [33] performed microarray analysis to assess medaka transcriptome modulation by NNV infection either followed by treatment with the antimicrobial peptides (AMPs) epinecidin-1(Epi-1) or tilapia hepcidin 1–5 (TH1-5) or not. Transcriptome analysis revealed altered expression of genes involved in B-, T- and mast cell activation, and adipocytokine signaling. Medaka infected with NNV exhibited up-regulation of PVALB, CEBPA, IFIM, IFN, IL-6st, NF-kB2, SOCS3, SP1, and TGFb1. Pre-treatment with epinecidin-1 or TH1-5 prevented these genes’ up-regulation. Treatment with Epi-1 increased expression of genes involved in adipocytokine signaling and B cell activation and decreased certain genes involved in mast or T-cell activation. Treatment with TH1-5 also decreased expression of the cytokine R gene and deregulated genes involved in adipocytokine signaling, B cell, mast cell, and T-cell activation in NNV-infected fish. Subsequent gene expression analysis for selected genes revealed that CCL4 and CYP2P3 expression was greatly enhanced at 8 and 4 h post-treatment with Epi-1 and NNV. Transcript levels of BD, PVALB, CEBPA, IL-6st, NF-kB2, SP1, TGFb1, and TNFa were decreased during treatment with Epi-1 in NNV infected medaka. Co-injection of TH1-5 and NNV decreased NNV mediated induction of BD, PVALB, CEBPA, IFIM, IFN, IL-6ST, NFkB2, SOCS3, SP1, and TGF-b1 at 8 h post-injection; however, expression of IFN, TGFb1, NF-kB2, and TNF-a was increased by TH1-5 treatment at seven days post-injection. Strong IL-6 induction was observed in fish infected with NNV. Based on the study results, the authors hypothesize that NNV infection of medaka may initially induce CEBPA expression, followed by expression of NF-kB and SP1, and ultimately an expression of IFN-b and BD (b-defensin) l. Moreover, expression of the IFIM1 gene (encoding an interferon transmembrane protein) also seems increased. During the medaka inflammatory response, TGF-b1 stimulates TNFa gene expression, and PVALB (Parvalbumin), IL-6st (encoding the IL-6 receptor), and SOC3 (encoding a cytokine inhibitor) genes are also induced. Treatment of NNV-infected medaka with Epi-1 inhibits the expression of TGF-b1 and TNFa and may down-regulate more immune genes, including BD, PVALB, CEBPA, IL-6ST, NF-kB2, and SP1. Epi-1 may induce CYP2P3, PTGR1, and IL-1b genes, which result in the inactivation of leukotriene B4, and reduce oxidoreductase activity. Finally, co-treatment with Epi-1 and NNV enhances expression of the CEBPD and CCL4 genes; CCL4 actives signal transduction via CCR5, thereby influencing chemokine expression. NNV-infected medaka treated with TH1-5 exhibited increased expression of NF-kB2, PVALB, TGFB1, and TNFa [33].

### 4.6. Cell Lines

In order to take a closer look at the effects of nodavirus in infected fish, several studies have focused on the transcriptome profiling of in vitro infected cells. Such investigations have been performed in brain cell lines of European sea bass (DLB-1 cell line) [43] and *Lateolabrax japonicus* (LJB cells) [40,51], in grouper kidney cells (GK cell line) [30], in European sea bass head-kidney leukocytes [37], and in Asian sea bass epithelial cells (SB cell line) [35]. Similar studies were performed in ZF-4 cells, an embryonic-derived zebrafish cell line [34], in striped snakehead fish cells (SSN-1) [36], and in orange-spotted grouper (*Epinephelus coioides*) fin cells [39]. All de-regulated DEGs in analyzed cell lines are summarized in Table 5. 

Chaves-Pozo et al. [43] performed a transcriptomic analysis in an NNV-infected European sea bass brain derived cell line (DLB-1). The authors suggested that the immune response, and more specifically the interferon pathway, is insufficient to overcome the viral susceptibility of the fish brain cells and to cease the progress of the infection. Instead, the cells undergo apoptosis upon stress response due to the viral infection 72 hpi. Differential expression analysis showed the up-regulation of immune-related genes (Toll-like receptors, IFN-pathway proteins, clusters of differentiation, suppressor of cytokine signaling, interleukins and their receptors, genes related to the cytotoxic cells and macrophages, transcription factors, major histocompatibility (MHC) I and II genes, TNF and related proteins), heat-shock proteins (HSP70, HSP90, small HSP, chaperonins and DNAJ families, TRIM family proteins) or proteins involved in the apoptosis (Fas, TRAF2, AIP, Bid, BAX, CASP3, CASP6, CASP7, CASP9, CASP10, CytC, DIABLO, or Apaf-1). At the same time, there was a characteristic down-regulation of many proteins involved in neuronal mechanisms (i.e., vesicle transport, Rho family of GTPases), cellular metabolism (GLUD1, CDD, HMOX1), cell cycle arrest, and cytoskeleton (MAP6). Interestingly, the down-regulation of proteins involved in vesicle formation and neuron transport implies that the specific mechanism plays a major role in the fish nervous system and its protection from NNV infection [43]. It is worth mentioning that the majority of DEGs in the DLB-1 cell line, including viperin (Atlantic cod, European sea bass, grouper), Mx (Senegalese sole, European sea bass, grouper), MDA5 (Senegalese sole), and ISG15 (Atlantic cod, European sea bass, Senegalese sole), are also found in the brain tissue of fish during in vivo experiments upon NNV-infection. 

As seen in the DLB-1 cell line, RGNNV infection of *L. japonicus* brain cells (LJB) resulted in immune response regulation and apoptotic-related proteins’ transcription (e.g., ISG15, IRFs, TLRs, HSPs) to assist the virus in escaping the host antiviral response. In the same study, the p53 signaling pathway was shown to be repressed by down-regulation of p53 and its downstream target genes (BAX, CASP8, CytC) and by up-regulation of its negative regulators MDM2 and MDM4 [40]. The p53 signaling pathway repression has a dual effect in the viral response by inhibiting the innate antiviral immunity and the type I IFN response, and the virus-infected cells apoptosis [120], thus promoting virus replication in RGNNV-infected LJB cells and facilitating the transmission of newly formed viral particles to other cells. Furthermore, the miRNAs which play important roles in the viral infection regulation were identified in LJB cells infected with RGNNV, highlighting the involvement of miRNAs in immune-related signaling pathways in the brain, including autophagy, mitophagy, and TGF-beta signaling pathways during the NNV infection [51]. In another study, miRNAs derived from a grouper fin (GF-1) cell line infected with RGNNV were also related to immune response, as well as transcription regulation, oxidation-reduction process, proteolysis, and regulation of the apoptotic process, while four differentially expressed miRNAs were found to promote virus replication [39]. Thus, miRNAs in different types of cells seem to play a crucial role in the immune response of the fish when infected with betanodavirus.

It is worth mentioning that when head-kidney leukocytes (HKLs) derived from European sea bass were incubated with DLB-1 and NNV-infected DLB-1 cell lines, the HLKs did not exhibit increased cell-mediated cytotoxic (CMC) activity in the infected DLB-1 cell line. When the gene expression was compared between the uninfected and the NNV-infected cell line, the derived transcriptomic profiles were very similar, and the comparing up-regulated genes in infected samples were related mainly to metabolism, translation, respiratory electron chain, and protein ubiquitination, while detection of immunity-related DEGs was very low. At the same time, sea bream HLKs showed significantly increased CMC activity against NNV-infected cells, and the authors suggested that these data highlight the reason for the European sea bass’ high susceptibility to nodavirus infections which escape the CMC activity [37].

As epithelial cells are included in the physical barrier of the fish body, the activation of response to NNV-infection could serve as the first defense line to stop further spreading of the disease and hence could play an important role in its development. In an NNV-infected Asian seabass epithelial cell line, a strong up-regulation of cytokines (CC chemokine, CXCL6, CXCL10), pro-inflammatory cytokines (IL17Fl, TNFa, TNFAIP3), and IFN chemokines (stimulator of interferon gene, IRF3, IFN-a1) were observed. Certain ISGs such as GVIN1, IFI27L2, ISG15, and viperin were also strongly induced at 48 hpi; an Mx protein was significantly induced at 24 and 48 hpi. An elevation on the VSG and RTP3 genes expression was detected as early as 6 hpi, which continued until 48 hpi when the highest increase of the HSP30 and HSP70 genes was also documented, suggesting that the fight of the disease starts at the early stages by strong activation of the innate immunity before the virus infects the rest of the fish organs [35]. 

It is clear by now that the IFN I pathway is crucial for combating the NNV-infection in fish; however, its stimulation has to be balanced in order to act in the host’s favor. In teleost fish, there are two types (type I and type II) of IFN I response, the first type of which (type I) is well studied and has been mentioned numerous times so far. Chen et al. [34] described the activation of both IFN I types after infecting a zebrafish cell line (ZF-4) with NNV. More specifically, the authors showed the up-regulation of both MyD88-dependent TLRs and RLRs signaling, with the latter regulating the IFN I pathway’s second group (type II) of proteins expression (up-regulation of MDA5, LGP2, TRAF3). The activation of the expression for MyD88, TRAF6, IRAK1 genes was related to MyD88-dependent TLR signaling, while the authors also displayed up-regulation of anti-viral proteins (IFNPHI1, IFNPHI3, MxA, Mxc, PKZ, ISG15) and transcription factors (STAT1A, STAT2, NFKB2) in NNV-infected ZF-4 cells [34].

The endoplasmic reticulum (ER) stress pathway was found to be primarily affected during the analysis of gene expression profiles in NNV-infected grouper kidney cells. More specifically, 16 UPR (unfolded protein response) and ER stress-associated genes, including BiP (immunoglobulin heavy-chain binding protein), PERK (PKR-like ER kinase), ATF6 (activating transcription factor 6), IRE1 (inositol-requiring enzyme 1), and XBP-1, were found to be affected during NNV infection. For instance, two components of the PERK pathway, namely CHOP and GADD34, were found to be up-regulated. Additionally, a major UPR regulator, BiP, as well as three BiP mediators, PERK, ATF6, and IRE1, were up-regulated at 33 hpi. Thus, the authors suggest that during an ER stress response against NNV-infection, the PERK pathway functions first while it was also shown that there is an interaction of BiP with two NNV proteins that is RdRp and capsid, with the latter having the ability to enter the nucleus of the infected cells in order to play a role in gene expression [30]. 

Apoptosis is another cell function that plays a fundamental role in fighting an infected host cell. In a striped snakehead fish (*Channa striatus*) cell line (SSN-1) infected with RGNNV, Chen et al. [36] identified DEGs related to viral pathogenesis, including RIG-I like receptors pathway, apoptosis pathway, oxidative phosphorylation, PI3K-Akt signaling pathway, and MAPK signaling pathway. The absence of alteration in the gene expression levels of TRAIL and TNF receptor-associated factor 1 and 2 genes in combination with the significant up-regulation of Endonuclease G (EndoG) at 3 and 24 hpi in SSN-1 cells indicate that the RGNNV infection potentially induces apoptosis of SSN-1 cells via the mitochondrial pathway instead of following the extrinsic pathway [36].

### 4.7. Pathway Analysis

**Figure 3:**
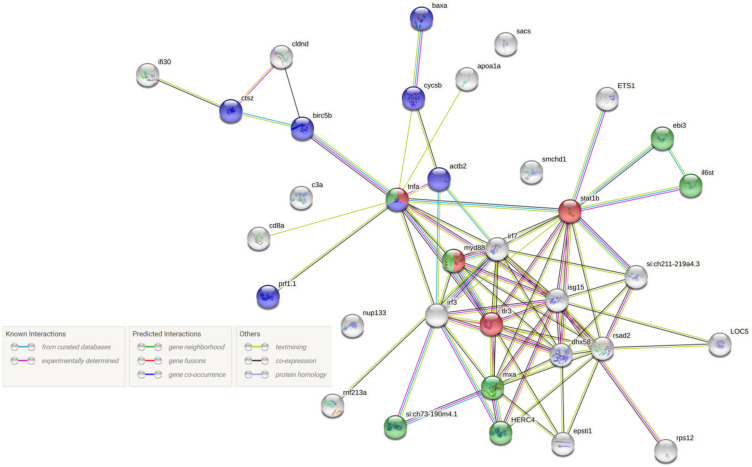
Protein-protein interaction (PPI) networks for the most frequently differentially expressed proteins found in different teleost fishes during NNV infection. This is retrieved via API access to the STRING database (https://string-db.org) (accessed on 14 January 2022) and was performed based on the *Danio rerio* protein database. Of the 65 proteins, 34 proteins are illustrated, as the rest were not found in the Search Tool for the Retrieval of Interacting Genes/Proteins database of the STRING database. Each node represents a differentially expressed protein. Node colors illustrate a protein’s involvement in the three significantly enriched biological processes identified by Biological Networks Gene Ontology (red nodes, Toll-like receptor signaling pathway; blue nodes, apoptosis; green nodes, cytokine signaling in the immune system). Gray nodes are not associated with any of the significantly enriched biological processes. The edges represent protein-protein interactions, and the nature of the protein-protein interactions are color-coded, as indicated in the figure.

Putting together the resulting data from different fish species, at different time points in the disease, it is now confirmed that the presence of betanodavirus in a host results in differentiation of expression of genes related mostly in innate immune signaling pathways, and specifically in IFN I pathway, in the apoptosis, and cell proliferation pathway. Moreover, virus responsive genes and genes related to antigen processing and presentation are also differentially expressed during the infection. Surprisingly, there seems to be a high consistency of DEGs and pathways in different fish tissues during the infection (brain, kidney, spleen, and liver). In Figure 2, the genes most frequently found to be differentially expressed in different fish are presented. In order to evaluate the ‘universal’ elements of host response based on potent enrichments of these genes in specific pathways, all genes annotated to zebrafish were subjected to analysis using the STRING database (https://string-db.org) (accessed on 14 January 2022). Hence, the 71 genes of *Danio rerio* (zebrafish) were analyzed in terms of the resulting reactome and KEGG pathways (Figure 3 and Figure S1). As expected, Toll-like receptor signaling pathways (4/89-red nodes) and apoptosis (7/181-blue nodes) emerged as the predominantly influenced KEGG pathways, while “cytokine signaling in the immune system” (7/295-green nodes) and the “immune system” (11/1364-yellow nodes) emerged in the reactome analysis. It is worth mentioning that TNFa is a link gene between the apoptosis (blue) and the TLR signaling (red) pathways, while it is also a member of cytokine signaling. Moreover, interferon regulatory factors 3 and 7 (IRF3, IRF7) also seem to act as connecting links between the two predominant KEGG-pathways highlighted by this analysis. 

## 5. Materials and Methods

### 5.1. Search Strategy

The present review followed guidelines set by the Preferred Reporting Items for the Systematic Reviews and Meta-Analyses (PRISMA) [121]. Several searches were performed with the keywords “transcriptome AND nervous necrosis virus”, “transcriptome AND betanodavirus”, “microarrays AND nervous necrosis virus”, and “transcriptome AND betanodavirus”. The following electronic databases were accessed repeatedly from September 2020 to May 2021: PubMed, Web of Science, SCOPUS, and Google scholar. Only accepted article publications were included in the present review (meeting abstracts and review articles were excluded), and though there was no restriction by language, all retrieved articles were in English.

### 5.2. Study Selection Process and Eligibility Criteria

Two independent researchers evaluated the full text of potentially eligible references and excluded those that were not met the following selection criteria: (1) the article had to be original; thus, all reviews were excluded, (2) the studied species should be fish; therefore crustacean and other species were excluded, (3) both in vivo studies and cell cultures with experimental or natural infections were included, (4) all expression studies contained data from infected versus non-infected fish (control), and (5) the article had to include significant changes in gene expression (data already analyzed by the authors).

### 5.3. Data Extraction

Editorial and experimental information from the included articles were summarized in Tables S1 and S2. Table S1 included the following items: article title, authors’ information, i.e., corresponding authors names, year of publication, country where the study was performed, journal where the article was published, study subject (fish species, developmental stage, and mean body weight), information of nervous necrosis virus type and experimental design (infection dose and type, sampling time points, and tested organ(s) or cell culture types). Table S2 contained information on the microarray/sequencing data of each article. It included the following items: data repository accession number of the article dataset, analysis platform and manufacturer, RNA isolation method, cDNA library preparation method, analyzed species, pooling, differential expressed genes (DEGs) criteria and statistical test, and finally, the use of housekeeping gene for qPCR validation. The data extraction process was conducted by two researchers in parallel. Any data discrepancies were solved by a third reviewer, as suggested by Caruffo et al. [52].

### 5.4. Gene Name Standardization

To determine the commonly de-regulated genes in response to infection, all DEGs were extracted from the main body text of the studies included in this review. To compare shared genes, gene abbreviations were converted to UniProt identification codes manually in the UniProt database (https://www.uniprot.org/) (accessed on 14 January 2022).

### 5.5. Phylogenetic Analysis

The phylogenetic analysis was based on cytochrome b [122]. Multiple alignments were conducted by MAFFT (Kazutaka Katoh, Japan, version: 7.490) [123]. Phylogenetic and molecular evolutionary analyses were conducted using MEGA version X [124]. The phylogenetic tree was constructed with the Neighbor-Joining method, using JTT with Freqs. (+F) model and gaps were removed by partial deletion. The topological stability was evaluated with 1000 bootstraps.

### 5.6. Construction of the PPI Network

DEGs found in more than one species were converted in *Danio rerio* corresponding genes. The Search Tool for the Retrieval of Interacting Genes/Proteins database (STRING v11.5) was subsequently used to construct their PPI network. Given a list of the proteins as input, STRING found their neighboring interactors and generated the PPI network consisting of all these proteins and all the interactions between them. All the interactions between them were derived from high-throughput lab experiments and previous knowledge in curated databases at a high level of confidence (sources: experiments, databases; score ≥ 0.90).

## 6. Conclusions

Disease management in the aquaculture industry is one of the greatest challenges; therefore, elucidation of host defense mechanisms to infectious pathogens such as NNV is crucial. Several studies have been published in the field of transcriptomic analysis to investigate gene expression responses to NNV infection in fish. Based on the results discussed above, universal host mechanisms against NNV infection became apparent, with interferon pathway key molecules playing the leading role. Genes including Mx, ISG15, ccl4, RTP3, Sacsin, GVINP1, IRF3, MDA5, VIPERIN, TRIM39, C3, CTSZ, CCL19, CD8, IFI44, IL12, IL8, ISG12, DHX58, MHC class Ia, MyD88, RNF213, STAT1, and TLR3 are repeatedly found in different hosts and conditions in the above mentioned studies and recent studies of our research group (manuscript under preparation), reflecting the core immune responsive DEGs following NNV infection. Several steps are still required to improve our understanding of NNV effects on host immune systems. Transcriptome analysis of VNN resistant and susceptible species or families of the same fish species, analysis of secondary lymphoid organs such as thymus and gills (GIALT), as well as virus target organs as eyes and experimental infection on long terms studies (>7 days post-infection), will shed light in our knowledge on NNV and host interaction.

Further studies are required, although some of the above mentioned genes could be used in the future as interspecies transcript biomarkers for assessing NNV-depended responses. In conclusion, even though transcriptome studies of NNV infected fish are increasingly oriented in elucidating molecules as biomarkers for specific functions, much more emphasis has to be given to exploring universal host reaction mechanisms, which will provide us with new perspectives in the battle of NNV infection to build healthier and sustainable aquaculture systems. 

## Figures and Tables

**Table 1:** Summary of fish species, analyzed organ, sampling time point, transcriptome analysis method, and the number of up- and downregulated DEGs of the eligible publications analyzed in the present review. N/A: non applicable.

Fish Species	Organ Sample/Cell Culture	Sampling Time	Analysis Method	Number of Up-Regulated DEGs	Number of Down-Regulated DEGs	Reference
*Psetta maxima*	Head kidney	3 hpi; 6 hpi; 24 hpi; 3 dpi	Microarray	94	12	[29]
*Epinephelus* spp.	Grouper kidney cells	6 hpi; 33 hpi	RNA-Seq	N/A	N/A	[30]
*Epinephelus coiodes*	Larvae whole	natural infection	Microarray	17	47	[31]
*Gadus morhua*	Brain	5 dpi; 11 dpi; 25 dpi	Microarray	1179	454	[32]
*Oryzias latipes*	Whole fish	24 hpi	Microarray	N/A	N/A	[33]
*Danio rerio*	Embryonic-derived zebrafish cells	6 hpi	Microarray	N/A	N/A	[34]
*Lates calcarifer*	Epithelial cells	6 hpi; 12 hpi; 24 hpi; 48 hpi	RNA-Seq	N/A	N/A	[35]
*Channa striatus*	Striped snakehead fish cells	3 hpi; 24 hpi	RNA-Seq	1184, 3 hpi 1138 24 hpi	1456, 3 hpi 2073, 24 hpi	[36]
*Dicentrarchus labrax*	Head-kidney leucocytes	4 hpi	RNA-Seq	N/A	N/A	[37]
*Hyporthodus septemfasciatus*	Brain	3 dpi; 4 dpi	RNA-Seq	N/A	N/A	[38]
*Epinephelus coiodes*	Grouper fin cell line	3 hpi; 24 hpi	RNA-Seq	43	8	[39]
*Lateolabrax japonicus*	*Lateolabrax japonicus* brain cells	48 hpi	RNA-Seq	1969	9858	[40]
*Solea senegalensis*	Head kidney; eye/Brain	48 hpi	RNA-Seq	N/A	N/A	[41]
*Epinephelus malabaricus*	Brain	9 dpi	RNA-Seq	N/A	N/A	[42]
*Dicentrarchus labra*	*D. labrax* brain cells	12 hpi; 3 dpi	RNA-Seq	834, 12 dpi 1053, 72 dpi	810, 12 dpi 1872, 12 dpi	[43]
*Epinephelus moara*	Liver; spleen; head kidney	natural infection	RNA-Seq	77, liver 89, spleen 318 kidney	6, liver 161, spleen 5314, kidney	[44]
*Dicentrarchus labrax*	Brain; head kidney	24 hpi; 3 dpi	RNA-Seq	N/A	N/A	[45]
*Dicentrarchus labrax*	Brain; head kidney	24 hpi; 3 dpi	RNA-Seq	196, head kidney 800, brain	44, head kidney 193, brain	[46]
*Lates calcarifer*	Head kidney	2 dpi; 15 dpi	RNA-Seq	N/A	N/A	[47]
*Epinephelus coiodes*	Larvae whole	5 dpi	RNA-Seq	17,939	2526	[48]
*Dicentrarchus labrax*	Head kidney; Brain	1, 3, 5, 7 dpi	OpenArray^®^ chip	N/A	N/A	[6]
*Solea senegalensis*	Head kidney; eye/Brain	2 dpi; 3 dpi	OpenArray^®^ chip	N/A	N/A	[49]
*Sparus aurata*	Larvae whole	6 hpi; 12 hpi; 24 hpi; 48 hpi	RNA-Seq	148, 12 dpi	158, 12 dpi	[50]
*Lateolabrax japonicus*	*Lateolabrax japonicus* brain cells	12 hpi; 48 hpi	RNA-Seq	35, 12 dpi 52, 48 dpi	54, 12 dpi 50, 48 dpi	[51]

**Table 2:** Differentially expressed genes in fish kidney tissues.

Fish Species	Up-Regulated DEGs	Down-Regulated DEGs
***Ps. maxima***	Mx, IFI35, BiP, serum lectin isoform 4, serum-inducible protein kinase, ceruloplasmin, kininogen I, haptoglobin, thrombin, proteinase activated receptor 3, apolipoprotein A1	F-box only protein 25, 5-aminolevulinate synthase, phosphatidylinositol 4-kinase
***S. sole***	Mx, LPG2 (DHX58), ISG15, STAT1, HERC5, IFI44, IFIT-1, NUP133, TRIM21, MACPF, u-PAR, PARP14, EPSTI1, ISG12, RNF213, HERC4, MHC class II, ANXA3, RTP3, CCL19L1, SMCHD1, ACTB, GIG1, interferon-induced very large, GTPase 1, IRF3, IRF7, TLR3, MAGEL2, GILT, Herpes gp2 multi-domain protein, claudin-like protein ZF-A89, 4F2 cell-surface antigen heavy chain-like, cathepsin Z, BIRC5, rho GTPase-activating protein 11A, v-ets erythroblastosis virus E26 oncogene homolog 1, C-type lectins, MDA5, LITAF, SACS/SACSIN, TRIM39, CCL4, CCK, EBI3	COL1A2, RDH13, RPS12, LMAN1/ERGIC-53, C3, C9, CFHR3
***E. moara***	MHC class I, HLA-A, MR1, lysozyme, hepcidin	C3, C9, IgM, IgD, IgT/IgZ, perforin, IL1, IL2, IL8, IL11, IL12, IL15, C1, C7, C8, CCL4, CXCL13, CXCL10, CCL3, CCL19, CXCL12, CCL20, CCL25, CCR9, CXCR4, CXCR3, CXR1
***D. labrax***	Mx, ISG15, ISG12, RTP3, SACS/SACSIN, TRIM39, CYP11b1, STAR, CYP21a, CYP17a1, 3BHSD, CYP11a1, IFI56	CCL4, ERO1a, HYOU1, PDIA4, QORX, CXC114, VLIG
***L. calcarifer***	beclin-1, IP3R, CYBA, C3aR, NCF2, OSBP1, cathepsin C/CTSC, IL5Rα/CD125	CD53, MHC class Ia, CH25H, BTK, STAT5b, SOCS1

**Table 3:** Differentially expressed genes in fish brain tissues.

Fish Species	Up-Regulated DEGs	Down-Regulated DEGs
***G. morhua***	LPG2 (DHX58), ISG15, IL8, MHC class Ia, NILT, NITR, CD3, CD8, TCR, RHOG1, ITK, FYB, LCK, granzyme, CC chemokines type-3, IL17a, MHC I complex-B2M, tapasins	
***S. sole***	Mx, LPG2 or DHX58, ISG15, STAT1, HERC5, IFI44, IFIT-1, NUP133, TRIM21, MACPF, u-PAR, PARP14, EPSTI1, ISG12, RNF213, HERC4, MHC class II, ANXA3, RTP3, CCL19L1, SMCHD1, ACTB, GIG1, interferon-induced very large GTPase 1, IRF3, IRF7, TLR3, MAGEL2, GILT, Herpes gp2 multi-domain protein, claudin-like protein ZF-A89, 4F2 cell-surface antigen heavy chain-like, cathepsin Z, BIRC5, rho GTPase-activating protein 11A, v-ets erythroblastosis virus E26 oncogene homolog 1, MDA5, SACS/SACSIN, TRIM39, MyD88, TBKBP1/SINTAD, PKR, HERC5	COL1A2, RDH13, RPS12
***H. septemfasciatus***	Mx, IFI44, CCL4, CXCL13, CCL19, CCL2, CCL34, CXCL6, CXCL8, CXCL9, IL12A, IL12B, IL18B, cathepsin L, cathepsin H, cathepsin K, cathepsin O, cathepsin S, cathepsin Z, CLEC4M, CLEC10A, CD209, LGALS9, LGALS3, FUCL4, MBL, RSAD2/VIPERIN, NK-Lysin, IFIT5, GVINP1, EIF2Ak2, IFIH1, HSP30	
***E. malabaricus***	SACS/SACSIN, CCL19, GVINP1, PD-L1, LAG3, CD3E, CD4, CD8A, RNF213, TREX, ZNFX1	NPTN, SF3B1, EWSR1, NLRX1, GNG3, MGEA5
***D. labrax***	Mx, ISG15, RTP3, IRF3, TLR3, C-type lectins, SACS/SACSIN, TRIM39, C3, CCL4, EBI3, IL8, RSAD2/VIPERIN, CD4, LECT2, NKTR, PTX4, C1QT1, PRL, SL, SOMA, GH, GLHA, TSH, POMC, 5HT3E, GABA, cathepsin, TLR8, TLR21, TNFa, IL1b, NCCRP-1, TCR-γ, CD8	IgM, STING, NLRC3

**Table 4:** Differentially expressed genes in fish larvae and whole fish.

Fish Species	Up-Regulated DEGs	Down-Regulated DEGs
***E. coioides***	apolipoprotein A1, MyBP-H, MLC2, white muscle troponin T, ADK1-2, MLC3, tropomyosin, parvalbumin PV1, parvalbumin PV2, trypsinogen, tubulin beta-1 chain, serine/threonine-protein phosphatase 5/AURKB, PAST-1, integrin alpha-V isoform X1, integrin beta-4, ANO10, chymotrypsinogen, claudin-15-like, afadin isoform X8, cadherin-17, collagen type I alpha 3 chains, collagen alpha-1(XVIII) chain isoform X2, collagen alpha-2(V) chain, 2-oxoglutarate 5-dioxygenase 1 isoform X2	C5, retinol-binding protein 4-A like, HEAT repeat-containing protein 1, importin-9
***S. aurata***	HSPA5, HSPA9, HSC70, RAB10, RAB11a, AP2a1, CHMP2a, TRAP1, UNC45b, FKBP4, PTGES3a, PEX5la, ATG4, ATG16, BNIP3, DPCP1	MyD88, IL12b, DNAJ4, IRF5, PIK3R1, STAT3, JAK1, IL6st, PEX3, PEX26, PEX11, EPHX2, ACOT8, GSTK1, LC3, ATG9L, STX17
***O. latipes***	PVALB, CEBPA, IFIM, IFN, IL-6ST, NF-kB2, SOC3, SP1, TGFB1	

**Table 5:** Differentially expressed genes in cell lines.

Fish Species	Up-Regulated DEGs	Down-Regulated DEGs
***D. labrax***	Mx, ISG15, MDA5, RSAD2/VIPERIN, HSP70, HSP90, Fas, TRAF2, AIP, Bid, Bax, CASP3, CASP6, CASP7, CASP9, CASP10, CytC, DIABLO, Apaf-1	GLUD1, CDD, HMOX1, MAP6
***L. japonicus***	ISG15, MDM2, MDM4	Bax, CytC, CASP8
***L. calcarifer***	Mx, RTP3, IRF3, CXCL10, CXCL6, RSAD2/VIPERIN, HSP30, TNFa, HSP70, CC chemokine, IL17Fl, TNFAIP3, IFN-a1, GVIN1, IFI27L2, VSG	
***D. rerio***	ISG15, STAT1, MDA5, MyD88, NF-kB2, LGP2, TRAF3, TRAF6, IRAK1, IFNPHI1, IFNPHI3, MxA, MxC, PKZ, STAT2	
***E.* spp.**	BiP, PERK, ATF6, IRE1, XBP-1, CHOP, GADD34	
***C. striatus***	Endonuclease G	

## Data Availability

Not applicable.

## Supplementary Materials

The following are available online at https://www.mdpi.com/article/10.3390/pathogens11020201/s1, Figure S1: Protein-protein interaction (PPI) networks for the most frequently differentially expressed proteins found in different teleost fishes during NNV infection including an additional enriched biological process (Immune system). Table S1: Eligible publications information (title, corresponding author, year of publication, country of origin, journal, fish species, fish development state/mean body weight, nervous necrosis virus type, infection dose, infection type, time post infection, organ sample/cell culture). Table S2: Transcriptome analysis information (data repository, analysis platform, manufacturer, RNA isolation, cDNA libraries, species, pooling, DEG criteria, statistical test to DEGs, annotation, enrichment & pathway analysis methods, qPCR validation). Table S3: Abbreviations.
