# Supplementary material for: Transcriptomic Analysis of Fish Hosts Responses to Nervous Necrosis Virus
Source: Pathogens. 2022 Feb 3;11(2):201. doi: 10.3390/pathogens11020201 (PMC8875540; doi:10.3390/pathogens11020201)
Supplement: Supplementary file 1 [file pathogens-11-00201-s001.zip › Toubanaki DK Manuscript_Dec21_SI.pdf]

Review

Transcriptomic Analysis of Nervous Necrosis Virus Interactions with Fish Hosts

Dimitra K. Toubanaki [1,*], Antonia Efstathiou [1] and Evdokia Karagouni [1,*]

Supplementary Material

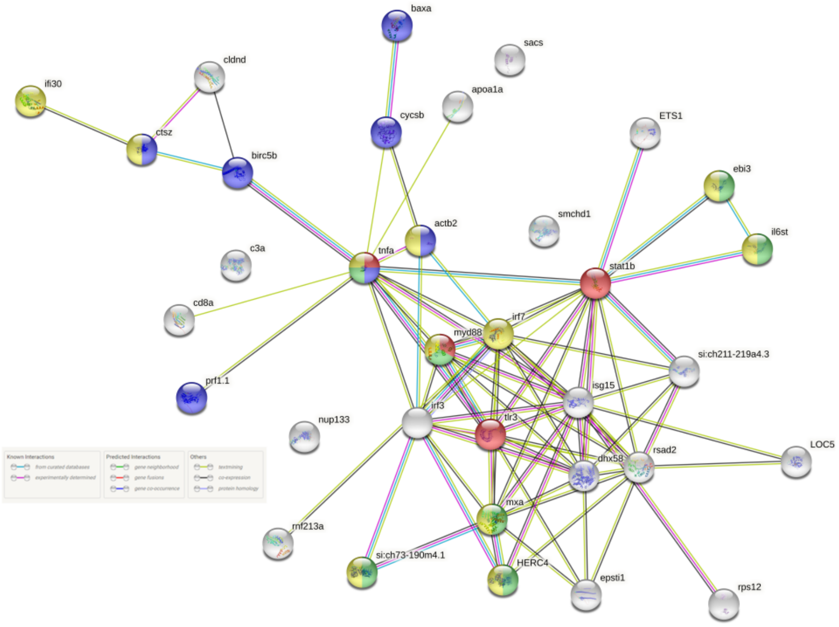

Figure S1: Protein-protein interaction (PPI) networks for the most frequently differentially expressed proteins found in different teleost fishes during NNV infection including an additional enriched biological process (Immune system). This is retrieved via API access to the STRING database (https://string-db.org) and was performed based on the Danio rerio protein database. Of the 65 proteins, 34 proteins are illustrated as the rest were not found in the Search Tool for the Retrieval of Interacting Genes/Proteins database of the STRING database. Each node represents a differentially expressed protein. Node colors illustrate a protein's involvement in the three significantly enriched biological processes identified by Biological Networks Gene Ontology (Red nodes: Toll-like receptor signalling pathway. Blue nodes: apoptosis. Green nodes: cytokine signalling in immune system. Yellow nodes: Immune system). Gray nodes are not associated with any of the significantly enriched biological processes. The edges represent protein-protein interactions, and the nature of the protein-protein interactions are color-coded as indicated in the figure.

Table S3. Abbreviations

**ACOT8**	Acyl-CoA Thioesterase 8
**ACNNV**	Atlantic Cod Nervous Necrosis Virus
**ACTB**	Actin Beta

**ACTH**	Adrenocorticotropic Hormone
**ADK1-2**	Adenylate Kinase 1- 2
**AIP**	Aryl Hydrocarbon Receptor-Interacting Protein
**AMPs**	Antimicrobial Peptide
**ANO10**	Anoctamin-10
**ANXA3**	Annexin A3
**Apaf-1**	Apoptotic Protease Activating Factor 1
**ApoA-I**	Apolipoprotein A1
**AP2a1**	Adaptor Related Protein Complex 2 Subunit Alpha 1
**ATF6**	Activating Transcription Factor 6
**ATG4**	Autophagy-Regulating Protease 4
**ATG9L**	Autophagy-Related Protein 9
**ATG16**	Autophagy Related Protein 16
**ATP2A1**	Sarcoplasmic Endoplasmic Reticulum Calcium Atpase 1-Like
**AURKB**	Serine/Threonine-Protein Phosphatase 5 Or Aurora Kinase B
**BAX**	Bcl2 Associated X, Apoptosis Regulator
**BD**	B-Defensin
**BFNNV**	Barfin Flounder Nervous Necrosis Virus
**Bid**	Bh3 Interacting Domain Death Agonist
**BiP**	Saxitoxin Binding Protein/Immunoglobulin Heavy-Chain Binding Protein
**BIRC5**	Baculoviral Iap Repeat Containing 5
**BLAST**	Basic Local Alignment Search Tool
**BNIP3**	BCL2/Adenovirus E1B 19 Kda Protein
**BP**	Biological Process
**B4GALT1**	Beta-1,4-Galactosyltransferase 1
**BTK**	Tyrosine-Protein Kinase
**C1**	Complement Component 1

**C1QT1**	Complement C1q Tumour Necrosis Factor-Related Protein 1
**C3**	Complement Component 3
**C3aR**	C3a Anaphylatoxin Chemotactic Receptor
**C5**	Complement Component 5
**C7**	Complement Component 7
**C8**	Complement Component 8
**C9**	Complement Component 9
**CARD**	Caspase Recruitment Domain
**CAMs**	Cell Adhesion Molecules
**CASP3**	Caspase 3
**CASP6**	Caspase 6
**CASP7**	Caspase 7
**CASP8**	Caspase 8
**CASP9**	Caspase 9
**CASP10**	Caspase 10
**CBLN1**	Cerebellin 1
**CC**	Cellular Component
**CCK**	Cholecystokinin
**CCL2**	C-C Motif Chemokine Ligand 2
**CCL3**	C-C Motif Chemokine Ligand 3
**CCL4**	C-C Motif Chemokine Ligand 4
**CCL19**	Chemokine (C-C Motif) Ligand 19
**CCL19L1**	Chemokine (C-C Motif) Ligand 19 L1
**CCL20**	C-C Motif Chemokine Ligand 20
**CCL25**	C-C Motif Chemokine Ligand 25
**CCR5**	C-C Chemokine Receptor Type 5
**CCR9**	C-C Motif Chemokine Receptor 9

**CDD**	Cytidine Deaminase
**cDNA**	Complementary DNA
**CD3E**	Cluster Of Differentiation 3 Epsilon
**CD8A**	Cluster Of Differentiation 8 Aplha
**CD3**	Cluster Of Differentiation 3
**CD4**	Cluster Of Differentiation 4
**CD8**	Cluster Of Differentiation 8
**CD28**	Cluster Of Differentiation 28
**CD53**	Leukocyte Surface Antigen
**CD125**	Cluster Of Differentiation 125
**CD209**	Cluster Of Differentiation 209
**CEBPA**	Ccaat Enhancer Binding Protein Alpha
**CEBPD**	Ccaat Enhancer Binding Protein Delta
**CFHR3**	Complement Factor H-Related Protein 3 Precursor)
**CHMP2a**	Charged Multivesicular Body Protein 2a
**CHOP**	C/EBP Homologous Protein
**CH25H**	Cholesterol 25-Hydroxylase
**circRNA**	Circular RNA
**CLEC4M**	C-Type Lectin Domain Family 4 Member M
**CLEC10A**	C-Type Lectin Domain Containing 10a
**CMC**	Cell-Mediated Cytotoxic
**CNS**	Central Nervous System
**COGs**	Clusters Of Orthologous Groups
**COL1A2**	Collagen Type I Alpha 2 Chain
**COX-2**	Cyclooxygenase 2
**CP**	Ceruloplasmin
**CTL**	Cytotoxic T Lymphocytes

**CUL1a**	Cullin 1a
**CXCL6**	C-X-C Motif Chemokine Ligand 6
**CXCL8**	C-X-C Motif Chemokine Ligand 8
**CXCL9**	C-X-C Motif Chemokine Ligand 9
**CXCL10**	C-X-C Motif Chemokine Ligand 10
**CXCL12**	C-X-C Motif Chemokine Ligand 12
**CXCL13**	C-X-C Motif Chemokine Ligand 13
**CXCL14**	C-X-C Motif Chemokine Ligand 14
**CXCR1**	C-X-C Motif Chemokine Receptor 1
**CXCR3**	C-X-C Motif Chemokine Receptor 3
**CXCR4**	C-X-C Motif Chemokine Receptor 4
**CXR1**	C-X-C Motif Chemokine Receptor 1
**CYBA**	Cytochrome B-245 Light Chain
**CYP2P3**	Cytochrome P450 2P3
**CYP11A1**	Cholesterol Side-Chain Cleavage Enzyme
**CYP11b1**	Cytochrome P450 11b
**CYP17a1**	Cytochrome P450 17a1
**CYP21A**	Steroid 21-Hydroxylase
**CytC**	Cytochrome C
**DAVID**	Database For Annotation, Visualization And Integrated Discovery
**DC**	Dendritic Cell
**DEGs**	Differentially Expressed Genes
**DHX58**	Laboratory Of Genetics And Physiology 2
**DIABLO**	Apoptotic Protease Activating Factor 1
**DLB-1**	D. Labrax Brain Cell Line
**DNA**	Deoxyribonucleic Acid
**DPCP1**	Mrna-Decapping Enzyme 1A

EBI3 Epstein-Barr Virus Induced 3

ECM Extracellular Matrix

EIF2Ak2 Eukaryotic Translation Initiation Factor 2 Alpha Kinase 2

EndoG Endonuclease G

EPHX2 Epoxide Hydrolase 2

Epi-1 Epinecidin-1

EPSTI1 Epithelial Stromal Interaction 1

ER Endoplasmic Reticulum

ERAP2 Endoplasmic Reticulum Aminopeptidase 2

ERGIC-53 ER-Golgi Intermediate Compartment 53 Kda Protein

ERO1a Endoplasmic Reticulum Oxidoreductase (Ero) 1-Like Protein Alpha

EWSR1 Ewing Sarcoma Protein (EWS) RNA Binding Protein 1

FAO Food And Agriculture Organization

Fas Fas Cell Surface Death Receptor

FBXl18 F-Box And Leucine Rich Repeat Protein 18

FKBP4 Peptidyl-Prolyl Cis-Trans Isomerase (FKBP) Prolyl Isomerase 4

FUCL4 Fucolectin

FYB Fyn Binding Protein 1

GABA Gamma-Aminobutyric Acid

GADD34 Growth Arrest And DNA Damage-Inducible Protein

GALT Gut-Associated Lymphoid Tissue

GEO Gene Expression Omnibus

GF-1 Grouper Fin Cell Line

GH Growth Hormone

GiALT Gill-Associated Lymphoid Tissue

GIG1 Protein GIGAS CELL1

GILT Gamma-Interferon-Inducible Lysosomal Thiol Reductase

**GK**	Grouper Kidney Cells
**GLHA**	Gonadotropin
**GLUD1**	Glutamate Dehydrogenase 1
**GNG3**	G Protein Subunit Gamma 3
**GO**	Gene Ontology
**GPR21**	G Protein-Coupled Receptor 21
**GRP78**	Glucose-Regulated Protein 78
**GSEA**	Gene Set Enrichment Analysis
**GSTK1**	Glutathione S-Transferase Kappa 1
**GVINP1**	Gtpase, Very Large Interferon Inducible Pseudogene 1
**HECTD2**	Hect Domain E3 Ubiquitin Protein Ligase 2
**HERC4**	Hect And Rld Domain Containing E3 Ubiquitin Protein Ligase 4
**HERC5**	Hect And Rld Domain Containing E3 Ubiquitin Protein Ligase 5
**HK**	Head Kidney
**HKLs**	Head-Kidney Leukocytes
**HLA-A**	Human Leukocyte Antigen A
**HMOX1**	Heme Oxygenase 1
**HPI**	Hypothalamic-Pituitary-Interrenal
**HSC70**	Heat Shock Cognate 71 Kda Protein
**HSP**	Heat Shock Protein
**HSPA5**	Heat Shock Protein 5
**HSPA9**	Heat Shock Protein 9
**HSP30**	Heat Shock Protein 30
**HSP70**	Heat Shock Protein 70
**HSP90**	Heat Shock Protein 90
**HYOU1**	Hypoxia Up-Regulated Protein 1
**IFI35**	Interferon Inducible Protein 35

**IFI44**	Interferon Induced Protein 44
**IFI56**	Interferon Induced Protein 56
**IFI27L2**	Interferon Alpha Inducible Protein 27 Like 2
**IFIH1**	Interferon Induced With Helicase C Domain 1
**IFIM**	Interferon-Induced Transmembrane Protein
**IFIT-1**	Interferon Induced Protein With Tetratricopeptide Repeats 1
**IFIT5**	Interferon Induced Protein With Tetratricopeptide Repeats 5
**IFN**	Interferon
**IFN-I**	Interferon-I
**IFNPHI1**	Interferon Phi 1
**IFNPHI3**	Interferon Phi 3
**IgA**	Immunoglobulin A
**IgD**	Immunoglobulin D
**IgHM**	Immunoglobulin M Heavy Chain
**IgM**	Immunoglobulin M
**IgT**	Immunoglobulin T
**IgZ**	Immunoglobulin Z
**IL-1**	Interleukin 1
**IL-1b**	Interleukin 1 Beta
**IL-2**	Interleukin 2
**IL-8**	Interleukin 8
**IL-10**	Interleukin 10
**IL-11**	Interleukin 11
**IL-12**	Interleukin 12
**IL-15**	Interleukin 15
**IL-17a**	Interleukin 15 Alpha
**IL5Rα**	Interleukin-5 Receptor Subunit Alpha

**IL6st**	Interleukin 6 Cytokine Family Signal Transducer
**IL12a**	Interleukin 12 Alpha
**IL12b**	Interleukin 12 Beta
**IL18b**	Interleukin 18 Beta
**ILF3/NF90**	Interleukin Enhancer Binding Factor 3
**IM**	Intramuscular Injection
**IP3R**	Inositol 1,4,5-Trisphosphate Receptor
**IRAK1**	Interleukin 1 Receptor Associated Kinase 1
**IRE1**	Inositol-Requiring Enzyme 1
**IRF**	Interferon Regulatory Factor
**IRF3**	Interferon Regulatory Factor 3
**IRF5**	Interferon Regulatory Factor 5
**IRF7**	Interferon Regulatory Factor 7
**IPA**	Ingenuity Pathway Analysis
**ISG**	Interferon Stimulated Gene
**ISG12**	Interferon-Stimulated Gene 12
**ISG15**	Interferon-Stimulated Gene 15
**ITGA2**	Integrin Subunit Alpha 2
**ITK**	Tyrosine-Protein Kinase
**ITGA6**	Integrin Subunit Alpha 6
**JAK1**	Janus Kinase 1
**JAK-STAT**	Janus Kinases - Signal Transducer And Activator Of Transcription Proteins
**KEGG**	Kyoto Encyclopedia Of Genes And Genomes
**KSNNV**	Korean Shellfish Nervous Necrosis Virus
**LAG3**	Lymphocyte Activating 3
**LAMA5**	Laminin Subunit Alpha 5
**LAMB2**	Laminin Subunit Beta 2

**LATS2**	Large Tumor Suppressor Kinase 2
**LC3**	Microtubule-Associated Protein 1A/1B-Light Chain 3
**LCK**	Lymphocyte-Specific Protein Tyrosine Kinase
**LECT2**	Leukocyte Cell Derived Chemotaxin 2
**LGALS3**	Galectin 3
**LGALS9**	Galectin 9
**LGP2**	Laboratory Of Genetics And Physiology 2
**LJB**	Lateolabrax Japonicas Brain Cells
**LITAF**	Lipopolysaccharide Induced Tnf Factor
**LMAN1**	Lectin Mannose Binding 1
**lncRNA**	Long Non-Coding RNA
**LTPs**	Lipid Transfer Proteins
**MACPF**	Membrane Attack Complex/Perforin
**MAGEL2**	Melanoma Antigen Gene (Mage) Family Member L2
**MALT**	Mucosal-Associated Lymphoid Tissues
**MAPK**	Mitogen-Activated Protein Kinase
**MAP6**	Microtubule Associated Protein 6
**MBL**	Mannose-Binding Lectin
**MDA5**	Melanoma Differentiation-Associated 5 Gene
**MDM2**	Mdm2 Proto-Oncogene
**MDM4**	Mdm4 Regulator Of P53
**MF**	Molecular Function
**MGEA5**	Meningioma Expressed Antigen 5
**MHC**	Major Histocompatibility Complex
**MHC-I**	Major Histocompatibility Complex I
**MHC-II**	Major Histocompatibility Complex II
**miRNA**	Microrna

**MLC2**	Myosin Light Chain 2
**MLC3**	Myosin Light Chain 3
**MR1**	Major Histocompatibility Complex, Class I-Related
**mRNA**	Messenger RNA
**Mt**	Million Tons
**MTUB**	Tubulin/Microtubule
**mut**	Mutant Strain
**Mx**	Myxovirus Resistance Protein
**MxA**	Myxovirus Resistance Protein 1
**MxB**	Myxovirus Resistance Protein B
**Mxc**	Myxovirus (Influenza Virus) Resistance C
**MyBP-H**	H-Like Myosin Binding Protein
**Myd88**	Myeloid Differentiation Primary Response Gene
**NADPH**	Nicotinamide Adenine Dinucleotide Phosphate
**NCBI**	National Center For Biotechnology Information
**NCCRP-1**	Nonspecific Cytotoxic Cell Receptor Protein-1
**NCF2**	Neutrophil Cytosol Factor 2-Like
**ncRNA**	Non-Coding RNA
**NF-κB**	Nuclear Factor Kappa-Light-Chain-Enhancer Of Activated B Cells
**NGS**	Next Generation Sequencing
**NILT**	Novel Ig-Liketype Receptor
**NITR**	Novel Immune-Type Receptor
**NKTR**	Natural Killer Cell Triggering Receptor
**NKTRP**	Natural Killer Tumour Recognition Protein
**NLR**	Nucleotide-Binding Oligomerization Domain (NOD)-Like Receptor
**NLRC3**	NLR Family CARD Domain Containing 3
**NLRX1**	Nucleotide-Binding Oligomerization Domain, Leucine Rich Repeat Containing X1

**NMDE2**	Glutamate Receptor Ionotropic
**NMI**	N-Myc Interactor
**NNV**	Nervous Necrosis Virus
**NOS1**	Nitric Oxide Synthase 1
**NR**	NCBI Non-Redundant Protein
**NT**	NCBI Nucleotide Sequences
**NPTN**	Neuroplastin
**NUP133**	Nucleoporin 133
**ORA**	Over Representation Analysis
**OSBP1**	Oxysterol-Binding Protein 1
**PARP14**	Poly(Adp-Ribose) Polymerase Family Member 14
**PAST-1**	Putative Achaete Scute Target 1
**PDIA4**	Protein Disulfide-Isomerase A4
**PD-L1**	Programmed Death-Ligand 1
**PERK**	PKR-Like ER Kinase
**PEX3**	Peroxisomal Biogenesis Factor 3
**PEX11**	Peroxisomal Biogenesis Factor 11
**PEX26**	Peroxisomal Biogenesis Factor 26
**PKR**	Protein Kinase R
**PKZ**	Z-DNA Binding Protein Kinase
**PI3K-Akt**	Phosphatidylinositol-3-Kinase/ Protein Kinase B (Akt)
**PIK3R1**	Phosphoinositide-3-Kinase Regulatory Subunit 1
**piRNA**	Piwi-Interacting RNA
**POMC**	Pro-Opiomelanocortin
**PPAR**	Peroxisome Proliferator-Activated Receptor
**Prf1**	Perforin 1
**PRISMA**	Preferred Reporting Items For The Systematic Reviews And Meta-Analyses

**PRL**	Prolactin
**PRRs**	Pattern Recognition Receptors
**PTGR1**	Prostaglandin Reductase 1
**PTGER4**	Prostaglandin E Receptor 4
**PTGES3A**	Prostaglandin E Synthase 3 (Cytosolic)
**PTX4**	Pentraxin 4
**PVALB**	Parvalbumin
**PV1**	Parvalbumin 1
**PV2**	Parvalbumin 2
**QORX**	Quinone Oxidoreductase Pig3
**qPCR**	Quantitative PCR
**RAB10**	Ras-Related Gtp-Binding Protein
**RAB11A**	Ras-Related Protein Rab-11a
**RC3H2**	Ring Finger And Ccch-Type Domains 2
**RdRp**	RNA Dependent RNA Polymerase
**RDH13**	Retinol Dehydrogenase 13
**RGNNV**	Red-Spotted Grouper Nervous Necrosis Virus
**RGS6**	G-Protein Signalling 6-Like
**RhoG1**	Rho-Related GTP-Binding Protein 1
**RIG1**	Retinoic Acid-Inducible Gene-I
**RLR**	Retinoic Acid-Inducible Gene-I (RIG1)-Like Receptors
**RNA**	Ribonucleic Acid
**RNA-seq**	RNA Sequencing
**RNF213**	Ring Finger Protein 213
**ROS**	Reactive Oxygen Species
**RPS12**	Ribosomal Protein S12
**RSAD2**	Radical S-Adenosyl Methionin Domain-Containing Protein 2

**RTP3**	Receptor Transporter Protein 3
**SAA**	Serum Amyloid A
**SACS**	Sacsin Molecular Chaperone
**SALT**	Skin-Associated Lymphoid Tissue
**SB**	Asian Sea Bass Epithelial Cells
**SEMA4B**	Semaphorin 4B
**SERCA1**	Sarcoplasmic Endoplasmic Reticulum Calcium Atpase 1-Like
**SF3B1**	Splicing Factor 3b Subunit 1
**SJNNV**	Striped Jack Nervous Necrosis Virus
**siRNA**	Small Interfering RNA
**SL**	Somatolactin
**SMCHD1**	Structural Maintenance Of Chromosomes Flexible Hinge Domain Containing 1
**snRNA**	Small Nuclear RNA
**snoRNA**	Small Nucleolar RNA
**SOCS1**	Suppressor Of Cytokine Signaling 1
**SOCS3**	Suppressor Of Cytokine Signaling 3
**SOMA**	Somatotropin
**Sox11-b**	SRY-Box Transcription Factor 11-B
**SP1**	Sp1 Transcription Factor
**SRA**	Sequence Read Archive
**SSN-1**	Striped Snakehead Fish Cells
**ssRNA**	Single Stranded RNA
**STAR**	Steroidogenic Acute Regulatory Protein
**STAT**	Signal Transducer And Activator Of Transcription
**STAT1**	Signal Transducer And Activator Of Transcription 1
**STAT2**	Signal Transducer And Activator Of Transcription 2
**STAT3**	Signal Transducer And Activator Of Transcription 3

**STAT5b**	Signal Transducer And Activator Of Transcription 5B
**STX17**	Syntaxin-17
**TBK1**	TANK-Binding Kinase 1
**STING**	Stimulator Of Interferon Genes Protein
**TBKBP1**	TBK Binding Protein
**TCR**	T-Cell Receptor
**TCR-γ**	T-Cell Receptor Gamma
**TGF-b**	Transforming Growth Factor Beta
**TH1-5**	Tilapia Hepcidin 1-5
**TLR**	Toll-Like Receptor
**TLR3**	Toll-Like Receptor 3
**TLR7**	Toll-Like Receptor 7
**TLR8**	Toll-Like Receptor 8
**TLR9**	Toll-Like Receptor 9
**TLR21**	Toll-Like Receptor 21
**TM**	Tropomyosin
**TNF-a**	Tumor Necrosis Factor Alpha
**TNFAIP3**	Tnf Alpha Induced Protein 3
**TnT**	Fast/White Muscle Troponin T
**TNV**	Turbot Nervous Necrosis Virus
**TPNNV**	Tiger Puffer Nervous Necrosis Virus
**TRAF2**	Tnf Receptor Associated Factor 2
**TRAF3**	Tnf Receptor Associated Factor 3
**TRAF6**	Tnf Receptor Associated Factor 6
**TRAIL**	TNF-Related Apoptosis-Inducing Ligand
**TRAP1**	Tnf Receptor Associated Protein 1
**Treg**	Regulatory T Cell

**TREX**	Three Prime Repair Exonuclease
**TRIM21**	Tripartite Motif Containing 21
**TRIM39**	Tripartite Motif Containing 39
**TSH**	Thyrotropin
**UNC45b**	Unc-45 Myosin Chaperone B
**u-PAR**	Urokinase Plasminogen Activator Surface Receptor
**UPR**	Unfolded Protein Response
**VAMP8**	Vesicle-Associated Membrane Protein 8
**VER**	Vacuolating Encephalopathy And Retinopathy
**viperin**	Radical S-Adenosyl Methionin Domain-Containing Protein 2
**VLIG**	Very Large Inducible GTPASE
**VNN**	Viral Nervous Necrosis
**VRC**	Viral Replication Complex
**VRG**	Virus Responsive Gene
**VSG**	Variant Surface Glycoprotein
**WDR55**	Wd Repeat Domain 55
**wt**	Wild Type
**XBP-1**	X-Box Binding Protein 1
**ZNFX1**	Zinc Finger Nfx1-Type Containing 1
**3BHSD**	Beta-Hydroxysteroid Dehydrogenase
**5HT3E**	Serotonin Receptor 5-Hydroxytryptamine Receptor 3E
**25HC**	25-Hydroxycholesterol
