# Supplementary figures and images for: Transcriptomic Analysis of Fish Hosts Responses to Nervous Necrosis Virus
Source: Pathogens. 2022 Feb 3;11(2):201. doi: 10.3390/pathogens11020201 (PMC8875540; doi:10.3390/pathogens11020201)

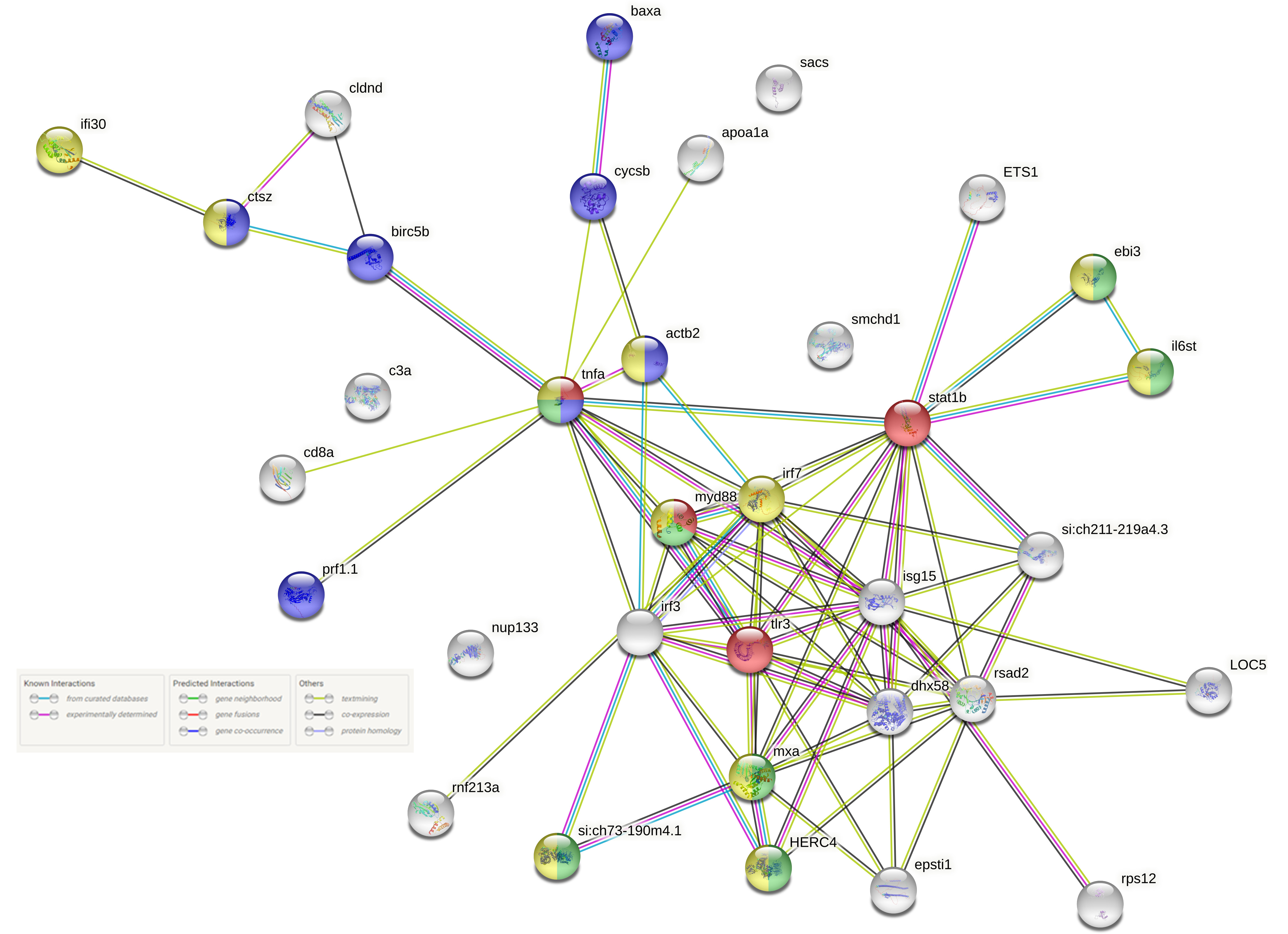

Supplement: Supplementary file 1 [file pathogens-11-00201-s001.zip › Figure S1.jpg]
